# Carbon nanotubes conjugated with cisplatin activate different apoptosis signaling pathways in 2D and 3D-spheroid triple-negative breast cancer cell cultures: a comparative study

**DOI:** 10.1007/s00204-024-03779-2

**Published:** 2024-05-13

**Authors:** Madalina Andreea Badea, Mihaela Balas, Daniela Ionita, Anca Dinischiotu

**Affiliations:** 1 Faculty of Biology, Department of Biochemistry and Molecular Biology, University of Bucharest, 91-95 Splaiul Independentei, 050095 Bucharest, Romania; 2 Research Institute of the University of Bucharest (ICUB), University of Bucharest, 90-92 Sos. Panduri, 050663 Bucharest, Romania; 3 Faculty of Applied Chemistry and Materials Science, Department of General Chemistry, Politehnica University of Bucharest, 313 Splaiul Independentei, 060042 Bucharest, Romania

**Keywords:** Cisplatin, Carbon nanotubes, Apoptosis, Breast cancer, Spheroids, Cell death

## Abstract

The type of experimental model for the in vitro testing of drug formulations efficiency represents an important tool in cancer biology, with great attention being granted to three-dimensional (3D) cultures as these offer a closer approximation of the clinical sensitivity of drugs. In this study, the effects induced by carboxyl-functionalized single-walled carbon nanotubes complexed with cisplatin (SWCNT–COOH–CDDP) and free components (SWCNT–COOH and CDDP) were compared between conventional 2D- and 3D-spheroid cultures of human breast cancer cells. The 2D and 3D breast cancer cultures were exposed to various doses of SWCNT–COOH (0.25–2 μg/mL), CDDP (0.158–1.26 μg/mL) and the same doses of SWNCT–COOH–CDDP complex for 24 and 48 h. The anti-tumor activity, including modulation of cell viability, oxidative stress, proliferation, apoptosis, and invasion potential, was explored by spectrophotometric and fluorometric methods, immunoblotting, optical and fluorescence microscopy. The SWCNT–COOH–CDDP complex proved to have high anti-cancer efficiency on 2D and 3D cultures by inhibiting cell proliferation and activating cell death. A dose of 0.632 μg/mL complex triggered different pathways of apoptosis in 2D and 3D cultures, by intrinsic, extrinsic, and reticulum endoplasmic pathways. Overall, the 2D cultures showed higher susceptibility to the action of complex compared to 3D cultures and SWCNT–COOH–CDDP proved enhanced anti-tumoral activity compared to free CDDP.

## Introduction

The first and most used cell culture system is the conventional two-dimensional (2D) cell culture, which involves growing cells on adherent plastic surfaces as monolayers. Besides the high contribution of 2D systems to the development of in vitro conditions to cultivate mammalian cells, the culture of cells as flat layers is associated with physiological changes and defined as a not-accurate model of in vivo cells (Antoni et al. 2015). In 2D cell culture, cells become flat and elongated, the gradients of nutrients, oxygen, and waste are absent, and as a result, cells receive the same amount of nutrients from the culture medium, and the gene and protein expressions are different compared to those of in vivo condition (Antoni et al. 2015; Caleb and Yong 2020).

In order to minimize these limitations and reproduce the three-dimensional (3D) organization of organisms, the 3D cultures were developed as cell systems that translate more accurately the in vivo environment. In 3D cultures, the natural cellular structure is preserved, the cells are heterogeneously exposed to culture media (the peripheral layer of cells is more exposed than the central layer), and cell–cell and cell–extracellular matrix interactions are predominant (Habanjar et al. 2021). Moreover, the responses of 3D cultures to radio- or chemotherapy are closer to the ones found in vivo, compared to 2D cultures (Kim et al. 2011). Among the many types of 3D tumor cell cultures (organoids, organ-on-a-chip, etc.), multicellular tumor spheroids (MCTSs) gained great attention and became an essential tool in cancer research and treatment. MCTSs are cell clusters with a heterogeneous structure that results from the gradients of nutrients and oxygen developed from the periphery to the spheroid center and the gradients of carbon dioxide and waste from the center to the spheroid border, resembling in vivo solid tumors (Han et al. 2021). The heterogeneous structure is based on a three-layered arrangement of proliferative, quiescent, and necrotic cells. The proliferative cells are organized in an outer layer, followed by an inner layer of quiescent cells and the necrotic cells are located in the center of the spheroid (Cui et al. 2017).

The MCTSs are models used to study various types of cancer, such as lung, pancreatic, liver, ovarian, prostate, and breast (Nayak et al. 2023). Among the breast cancer types, triple-negative breast cancer (TNBC) represents a priority in cancer treatment research due to its highly aggressive character and limited targeted treatment caused by the lack of HER2 overexpression and the absence of estrogen and progesterone receptors’ expression (Zagami and Carey 2022). The standard treatment options remain chemotherapy and surgery, with limited efficiency associated with high relapse and mortality, which triggers the exploration of different forms of therapy (Won and Spruck 2020; Han et al. 2023).

In the last era, nanoparticles (NPs) were studied as promising drug delivery systems for the transport of drugs to the tumor site due to their advantages in cancer treatment: reduced size, controllable structure, and tumor-targeted drug release (Shokooh et al. 2022). An emerging approach based on nanotechnology is the use of carbon nanotubes (CNTs) as a therapeutic route for TNBC. CNTs are cylindrical structures consisting of graphene, with lengths up to microns and a diameter of range in nanometers, which are classified into single- and multi-walled CNTs (SWCNTs and MWCNTs), depending on the number of layers of which they are made up. Moreover, CNTs are highly hydrophobic and require functionalization to improve their solubility in aqueous solutions before their use in biomedical applications (Rathinavel et al. 2021). The addition of functional groups on CNTs' surface improves water solubility and facilitates the complexation of other molecules or drugs with CNTs (Dubey et al. 2021). As nanocarriers, CNTs present important advantages over conventional therapeutic agents, such as targeted and controlled delivery at the tumor site, minimization of side effects, enhanced internalization and efficacy of antineoplastic agents, and delivery of combination of two or more drugs, which highlight their potential use as tools in cancer therapy (Grobmyer et al. 2011; Mendes et al. 2015).

Functionalized CNTs can be complexed with a high variety of chemotherapeutics including doxorubicin (Chadar et al. 2021), paclitaxel (Ghoderao et al. 2022), gemcitabine (Singh et al. 2013), methotrexate (Karimi et al. 2019), cisplatin (Badea et al. 2018), etc. Cisplatin (CDDP) is a platinum-based anti-cancer drug used in breast cancer treatment, which exerts its activity mainly by generating adducts with DNA and activating different signaling pathways that lead to cell death (Ghosh 2019). Other cellular targets of CDDP are RNA, mitochondrial DNA, proteins, membrane phospholipids, cytoskeletal microfilaments, and thiol-containing molecules (Gonzalez et al. 2001). The main cell death mechanism induced by CDDP is apoptosis (Tanida et al. 2012), but it can also trigger other forms of cell death, such as defective apoptosis or necrosis (Gonzalez et al. 2001).

In this context, our study aimed to compare the modulation of cell death signaling pathways in human breast cancer monolayer versus spheroid cultures under the action of carboxyl-functionalized single-walled carbon nanotubes conjugated with cisplatin (SWCNT–COOH–CDDP).

## Materials and methods

### Synthesis and characterization of SWCNT–COOH–CDDP complexes

Carboxyl-functionalized single-walled carbon nanotubes (SWCNT–COOH) were obtained by covalent functionalization of SWCNT through chemical oxidation. Further, SWCNT–COOH was functionalized with CDDP to obtain carboxyl-functionalized single-walled carbon nanotubes conjugated with CDDP (SWCNT–COOH–CDDP). The detailed procedure of synthesis and characterization of the complex is presented in a previous study (Badea et al. 2022).

### Cell culture and treatment conditions

Human MDA-MB-231 epithelial cell line (HTB-26^™^, ATCC, Manassas, VA), from an adenocarcinoma of the mammary gland, was grown as monolayer cultures in 75 cm^2^ flasks and Dulbecco’s Modified Eagle Medium (DMEM, 31,600-083, Gibco, UK) supplemented with 3.5 g/L glucose, 1.5 g/L NaHCO_3_, 1% penicillin/streptomycin/amphotericin B solution (A5955, Sigma-Aldrich, St. Louis, MO, USA), and 10% fetal bovine serum (10270-106, origin South America, Gibco, Life Technologies, Carlsbad, CA, USA). The culture medium was completely refreshed once every two–three days. For the subculture procedure, cells were detached from the flask surface using a 0.25% trypsin – 0.53 mM EDTA solution and the cells were counted using a Bürker-Türk chamber. The cultures were maintained in standard conditions (37 °C, humidity, 95% air, 5% CO_2_) and monitored every day.

MDA-MB-231 cells were seeded in culture plates and flasks and on the second day of culture were treated with various concentrations of CDDP (0.158, 0.316, 0.632, and 1.26 µg/mL), SWCNT–COOH (0.25, 0.5, 1 and 2 µg/mL), and the same doses of SWCNT–COOH–CDDP for 24 and 48 h. For treatment, the culture medium was removed and replaced with medium containing treatment of the tested concentrations. Untreated cells were used as control.

### MDA-MB-231 spheroids generation and treatment

Multicellular tumor spheroids (MCTSs) were generated from MDA-MB-231 cells in complete DMEM medium supplemented with 2.5% Matrigel (Matrigel Basement Membrane Matrix, 356237, Corning, NY) by liquid-overlay technique using low-cell-attachment Nunclon Sphera Microplates. Single MCTSs were generated in 96-well round bottom plates (cat. no. 12-566-430, 174925) in 200 µL culture medium and multiple MCTSs were obtained using 6-well plates (cat. no. 12-566-435, 174932) in 3 mL culture medium, according to our previous study (Badea et al. 2019).

MCTSs were seeded at a density of 5000 cells/well, respectively 5 × 10^5^ cells/well in 96- and 6-well plates. Starting with the third day of culture, MCTSs were exposed to the same doses of treatment as monolayers cultures for 24 and 48 h. For single MCTS, half of the culture medium was replaced with medium containing double concentrations of the tested samples. An identical procedure was followed for multiple MCTSs cultures, with the mention that the added medium contained Matrigel in a concentration of 1.25%. Untreated MCTSs represented the control.

### Lactate dehydrogenase (LDH) release assay

Membrane integrity and induction of cell death by necrosis pathway were evaluated by quantifying the level of lactate dehydrogenase (LDH) enzyme activity in the culture medium of 2D and 3D cultures. The method was performed using a commercial kit *Cytotoxicity Detection Kit (LDH)* purchased from Roche (cat. no. 11644793001) which used a catalyst (diaphorase/NAD^+^ mixture) and a dye solution (Iodotetrazolium chloride (INT) and sodium lactate) as reagents. A volume of 50 μL culture medium from treated and untreated cells and MCTSs was homogenized with 50 μL reaction mixture (catalyst:dye solution 1:45) and then incubated in the dark, at room temperature, for 15 min. The absorbance of samples was measured at 490 nm, at Flex Station 3 (Molecular Devices, San Jose, CA, USA). The results were expressed as percentages (%) of control (untreated cells and MCTSs respectively).

### Morphology assessment

After 24 and 48 h of treatment with the complex and free constituents, morphological changes of breast cancer cells and single MCTSs were investigated by optical microscopy using an inverted microscope Olympus IX73 (Olympus, Tokyo, Japan). The cells and MCTSs were imaged using a Hamamatsu camera (A3472-06, Hamamatsu, Japan) and CellSens Dimension software.

### Live/dead staining assay

To evaluate the cell viability after treatment exposure, treated cells and single MCTSs were fluorescently labeled with calcein AM and ethidium homodimer-1 (EthD-1), following the instructions of LIVE/DEAD^™^ Viability/Cytotoxicity Kit (L3224, Invitrogen). Calcein AM highlighted the live cells based on esterase activity and EthD-1 distinguished dead cells with a permeable membrane. Thus, the cells were seeded in 24-well plates (4 × 10^4^ cells/well) to form monolayer cultures and in Nunclon Sphera plates (5000 cells/well) for MCTSs generation. After 24 and 48 h from treatment, the culture medium of monolayer cultures was replaced with the reaction mix (a serum-free solution containing 2 μM calcein AM and 4 μM EthD-1). In parallel, half of the culture medium of single MCTSs was substituted with a similar solution containing double concentrations of the dyes. Afterward, monolayer cultures and MCTSs were incubated for 20 min, at 37 °C and then visualized at a fluorescence inverted microscope Olympus IX73 (Olympus, Tokyo, Japan). Images were acquired using FITC and TRITC filters and CellSens Dimension software (ver. 1.11, Olympus, Tokyo, Japan).

### Measurement of mitochondrial membrane potential (MMP)

Mitochondrial membrane potential (MMP) was evaluated after the treatment of 2D cultures and single MCTSs with two doses of CDDP (0.316 μg/mL and 0.632 μg/mL), SWCNT–COOH (0.5 and 1 μg/mL), and complex (same doses). For this assessment, the “Mitochondria Membrane Potential Kit” was used. This assay is based on a fluorometric method in which a decreased fluorescence is associated with apoptosis. In brief, MDA-MB-231 cells were seeded in 96-well plates to form 2D (1.2 × 10^4^ cells/well) and 3D (5000 cells/well) cultures and then treated with the samples. After 24 and 48 h, in monolayer cultures, the culture medium was discarded and the cells were incubated at 37 °C with 100 μL Dye Loading Solution for 30 min. Then, 50 μL Assay Buffer B was added to every well. After 30 min of incubation at 37 °C, the fluorescence was measured at Flex Station 3 (ex. 540 nm/em. 590 nm) and the results were calculated related to the control.

The same protocol was followed for 3D cultures, with the mention that 50 μL culture medium was kept in wells and then mixed with 50 μL Dye Loading Solution twice concentrated. Finally, the spheroids were analyzed under Olympus IX73 fluorescence microscope. The images were acquired using CellSens Dimension software and the Z-stack function. For each experimental condition, three spheroids were analyzed. Fluorescence level was quantified using ImageJ software (ver. 1.52a, NIH, Bethesda, MD, USA), indicated as corrected total cell fluorescence (CTCF) and represented related to control.

### Preparation of cell lysates and estimation of total protein content

For obtaining the cell lysates, MDA-MB-231 cells were seeded as 2D cultures in flasks (10^6^ cells/flask) and MCTSs in 6-well plates (5 × 10^5^ cells/well). After treatment with CDDP (0.316 and 0.632 μg/mL), SWCNT–COOH (0.5 and 1 μg/mL), and complex (same doses), the cells (enzymatically detached from the culture surface) and MCTSs were collected and centrifuged for 5 min, 1500 rpm. Cell pellets were washed and then resuspended in phosphate-buffered saline (PBS). Then, cells were lysed by sonication (30 s, 3 times, on ice) using a UP50H sonicator (Hielscher Ultrasound Technology, Teltow, Germany), set at 80% amplitude, 1 cycle. Cell lysates were centrifuged (10 min, 3000 rpm, 4 °C) and the supernatants were aliquoted, stored at – 80 °C, and used in the next week for biochemical analyses. Total protein content was quantitated by the Bradford assay using bovine serum albumin (BSA) standard curve (0–1.5 mg/mL) and spectrophotometric detection at 595 nm (Flex Station 3, Molecular Devices, San Jose, CA, USA) (Bradford 1976).

### Measurement of reduced glutathione (GSH) content

The antioxidant defense of 2D and 3D cultures after treatment was assessed by quantifying the concentration of reduced glutathione (GSH) using the reaction between GSH and Ellman's reagent—5,5ʹ-dithio-bis-(2-nitrobenzoic acid)—DTNB with the generation of 2-nitro-5-thiobenzoic (TNB) acid that can be spectrophotometrically detected at 405 nm. First, cell lysates were deproteinized with equal volumes of a 5% solution of 5-sulfosalicylic acid and centrifuged at 10,000 rpm, for 10 min, and 4 °C. Then, 10 μL of deproteinized lysate was homogenized with 150 μL reaction mix (1.5 mg/mL DTNB in potassium phosphate buffer 0.1 M, pH = 7 and EDTA 1 mM) and incubated for 10 min at room temperature. Finally, the absorbance was read at 405 nm using a Flex Station 3 microplate reader (Molecular Devices, San Jose, CA, USA). GSH concentration of the samples was estimated by extrapolation on a 3.125–200 μM GSH standard curve and calculated related to control (% of control).

### Western blot analysis

Western blot technique was used for the evaluation of various protein expressions to elucidate the mechanisms triggered in 2D cultures and spheroids of MDA-MB-231 cells after treatment with CDDP (0.316 and 0.632 μg/mL), SWCNT–COOH (0.5 and 1 μg/mL), and complex (same doses). The total protein extracts were diluted with PBS up to equal amounts of protein, and the samples were then subjected to a chemical and thermal (SDS, 5 min, 95 °C) denaturation. The proteins were separated on polyacrylamide gels (8, 10, 15%) in Tris-Gly electrophoresis buffer (0.05 M Tris, 0.05 M glycine, and 0.1% SDS), at 90 V, and then transferred onto polyvinylidene fluoride (PVDF) membranes (cat. no. IPVH00010, Merck, Darmstadt, Germany) using a Tris-Gly transfer buffer (25 mM Tris, 192 mM glycine, and 20% (v/v) methanol). The detection of protein bands was done using Western Breeze Chromogenic Anti-Mouse and Anti-Rabbit kits solutions (WB7103, WB7105, Invitrogen, Carlsbad, CA, USA). Following blocking of non-specific binding sites, the membranes were incubated overnight with primary antibody solutions anti-Nrf2 (sc-13032, Santa Cruz, CA, USA), anti-MCM2 (sc-373702, Santa Cruz), anti-caspase-3 (sc-7148, Santa Cruz), anti-caspase-8 (sc-5263, Santa Cruz), anti-caspase-9 (sc-56076, Santa Cruz), and anti-caspase-4 (sc-56056, Santa Cruz). On the next day, membranes were washed with wash solutions and incubated with anti-mouse and anti-rabbit secondary alkaline phosphatase-conjugated antibodies. Immune complexes were revealed with the BCIP/NBT chromogenic substrate and imaged using a ChemiDoc Imaging System (Bio-Rad, Hercules, CA, USA). Western blot signal intensities were quantified using the ImageLab program (version 6.1.0, Bio-Rad, Hercules, CA, USA). The β-actin protein served as a reference for normalization.

### Evaluation of invasion potential

The invasion capacity of MDA-MB-231 cells was assessed after the treatment of 2D and 3D cultures with CDDP (0.316 and 0.632 μg/mL), SWCNT–COOH (0.5 and 1 μg/mL), and the corresponding doses of the complex for 24 and 48 h.

In 2D cultures, the invasion potential was evaluated following the instructions of CytoSelect^™^ 96-Well Cell Invasion Assay (Basement Membrane, Fluorometric Format, CBA-112, Cell Biolabs), which uses a 96-well invasion plate (composed of feeder tray, membrane chamber, and plate cover) and a polycarbonate membrane inserts (8 µm pore size) to discriminate non-invasive cells from invasive cells which were detected with CyQuant^®^ GR dye. First, in the wells of the feeder tray were added 150 μL culture medium supplemented with 10% fetal bovine serum. In parallel, the membrane was rehydrated with 100 μL serum-free culture medium and after 1 h, the medium was replaced with 100 μL cell suspension (8 × 10^5^ cells/mL) prepared in free-serum culture medium supplemented with 5% BSA, also containing the treatment. Assays were carried out over 24 and 48 h of incubation in a humidified atmosphere of 5% CO_2_, and 95% air at 37 °C. The culture medium was removed from the membrane chamber, which was placed in the Cell Harvesting Tray containing 150 μL of Cell Detachment Solution/well and incubated for 30 min, at 37 °C. Then, in each well was added 50 μL of 4X Lysis Buffer/CyQuant^®^ GR dye solution and after 20 min of incubation at room temperature, the fluorescence was measured at Thermo Scientific Appliskan (Waltham, MA, USA), ex. 485 nm/em. 535 nm. The results were calculated related to control (% of control).

The cell invasion in 3D cultures was investigated after treatment using the manufacturer’s guidelines of “96-Well 3D Spheroid BME Cell Invasion Assay” (3500-096-K, Trevigen, Inc., Gaithersburg, MD). A matrix based on basement membrane proteins, which generates a hydrogel network that embeds the spheroid and can be penetrated by invasive cells, was used. Briefly, spheroids were seeded at a density of 3000 cells in 50 μL 1× Spheroid Formation ECM in 3D Culture Qualified 96-Well Spheroid Formation Plate. The plate was centrifuged at 200×g, 3 min, room temperature, to promote spheroid formation and then incubated at 37 °C. On the third day of culture, in the pre-chilled plate was added 50 μL of Invasion Matrix/well and the plate was centrifuged at 300×g, 4 °C for 5 min and then transferred in the incubator at 37 °C for 1 h to enhance gel formation. Further, culture media with or without indicated concentrations of treatment (100 μL/well) were added and then the plates were further incubated in a 5% CO_2_ atmosphere at 37 °C. After 24 and 48 h, the invasion of cells into the surrounding matrix was monitored, and the spheroids were photographed using a Hamamatsu camera (A3472-06, Hamamatsu, Japan) of an Olympus IX73 microscope (Olympus, Tokyo, Japan) and CellSens Dimension software (ver. 1.11, Olympus, Tokyo, Japan). Image analysis was performed using ImageJ software (ver. 1.52a, NIH, Bethesda, MD, USA) using the instructions of the kit. Five spheroids were analyzed for each experimental condition. Cell invasion was calculated by reporting the area of treated spheroids to the area of control spheroids (% of control).

### Statistical analysis

All investigations were performed in triplicate. The data were expressed as relative values in comparison with control (100%) and calculated as mean ± standard deviation. The results were statistically analyzed in GraphPad Prism (Version 8, GraphPad Software, La Jolla, CA, USA), using the two-way ANOVA method and Tukey’s multiple comparisons tests (treated cells vs. control). The values *p* < 0.05 (*), *p* < 0.01 (**), and *p* < 0.001 (***) were considered statistically significant.

## Results

### Anti-tumor efficiency of SWCNT–COOH–CDDP on 2D and 3D cultures

The anti-tumor efficiency of SWCNT–COOH–CDDP complex was assessed on 2D and 3D MDA-MB-231 cultures by evaluation of different parameters, such as lactate dehydrogenase (LDH) activity in culture medium, cell morphology, proliferation, and cell viability.

**Fig. 1:**
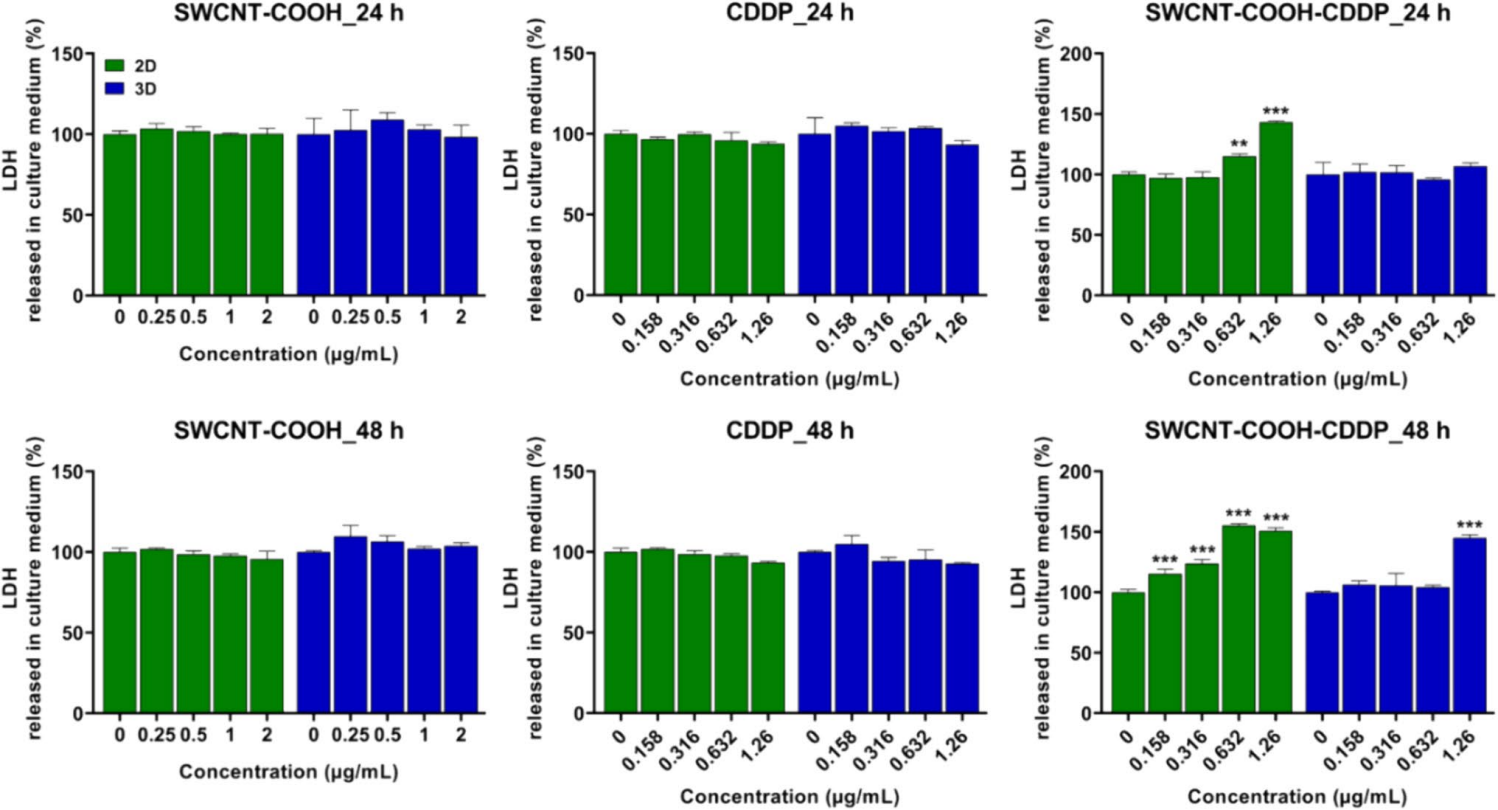
Relative level of lactate dehydrogenase (LDH) released in culture medium of 2D and 3D MDA-MB-231 cultures by CDDP (0.158–1.26 μg/mL), SWCNT–COOH (0.25–2 μg/mL) and SWCNT–COOH–CDDP (0.158–1.26 μg/mL) after 24 and 48 h of treatment. The concentration of SWCNT–COOH in complex is the same as free SWCNT–COOH. Untreated cultures (0 μg/mL) were used as control. Results (control vs. sample) were considered significant when *p* < 0.05 (*), *p* < 0.01 (**), *p* < 0.001 (***). Error bars reflect the standard deviation

LDH assay (Fig. 1) revealed that the free components induced no changes in LDH release in the culture medium of 2D and 3D cultures compared to control. A time-dependent release of LDH was registered only after the treatment with SWCNT–COOH–CDDP suspension. However, the effects were more pronounced in 2D cultures compared to 3D-spheroid cultures. After 24 h, LDH leakage increased only in the case of 2D cultures after the treatment with the highest doses of SWCNT–COOH–CDDP, while in 3D cultures, no modifications were observed. After 48 h, extracellular LDH level increased in 2D cultures starting with the lowest dose of SWCNT–COOH–CDDP (0.158 μg/mL), the highest increase being induced by a dose of 0.632 μg/mL. In the case of spheroids, the only increase (by 44.97% compared to control) was registered after the treatment with the dose of 1.26 μg/mL SWCNT–COOH–CDDP.

**Fig. 2:**
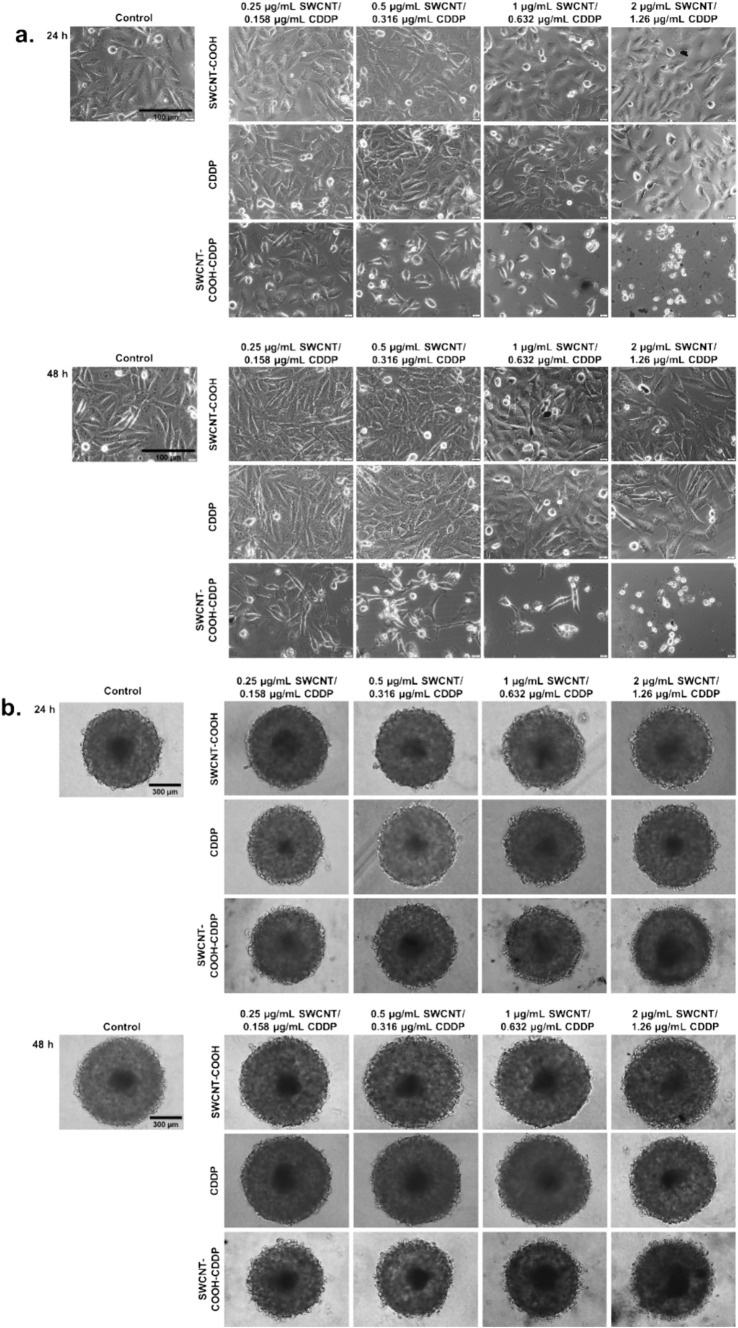
Optical microscopy images reflecting the morphology of MDA-MB-231 cells (**a**) and spheroids (**b**) after the treatment with CDDP (0.158–1.26 μg/mL), SWCNT–COOH (0.25–2 μg/mL) and SWCNT–COOH–CDDP (0.158–1.26 μg/mL) for 24 and 48 h. The concentration of SWCNT–COOH in complex is the same as free SWCNT–COOH. Control represented untreated cultures (0 μg/mL). Scale bar: 100 µm (**a**) and 300 µm (**b**)

The analyses of morphology revealed important alterations in both 2D and 3D cultures after the treatment with SWCNT–COOH–CDDP complex. MDA-MB-231 cells lost their specific characteristics and presented elongated morphology, vacuoles in the cytoplasm, cell shrinkage, and fragmentation starting with a dose of 0.316 μg/mL SWCNT–COOH–CDDP. Also, cell density was considerably reduced and cells became spherical in shape after the treatment with the highest two doses of the complex. After the treatment with free components, no changes in the cell morphology were observed except for a small reduction of cell density in the presence of 1.26 μg/mL CDDP after 24 and 48 h, respectively, as well as 2 μg/mL SWCNT–COOH after 48 h exposure (Fig. 2a).

After exposure of spheroids to the highest two doses of SWCNT–COOH–CDDP (0.632 and 1.26 μg/mL CDDP) for 24 h, the proliferative layer was affected and its dimensions were reduced. The same modifications were observed also after 48 h of exposure to all tested doses of SWCNT–COOH–CDDP. Exposure to CDDP and SWCNT–COOH induced no changes in spheroid morphology, except for the dose of 1.26 μg/mL CDDP when a low reduction of size was observed (Fig. 2b).

**Fig. 3:**
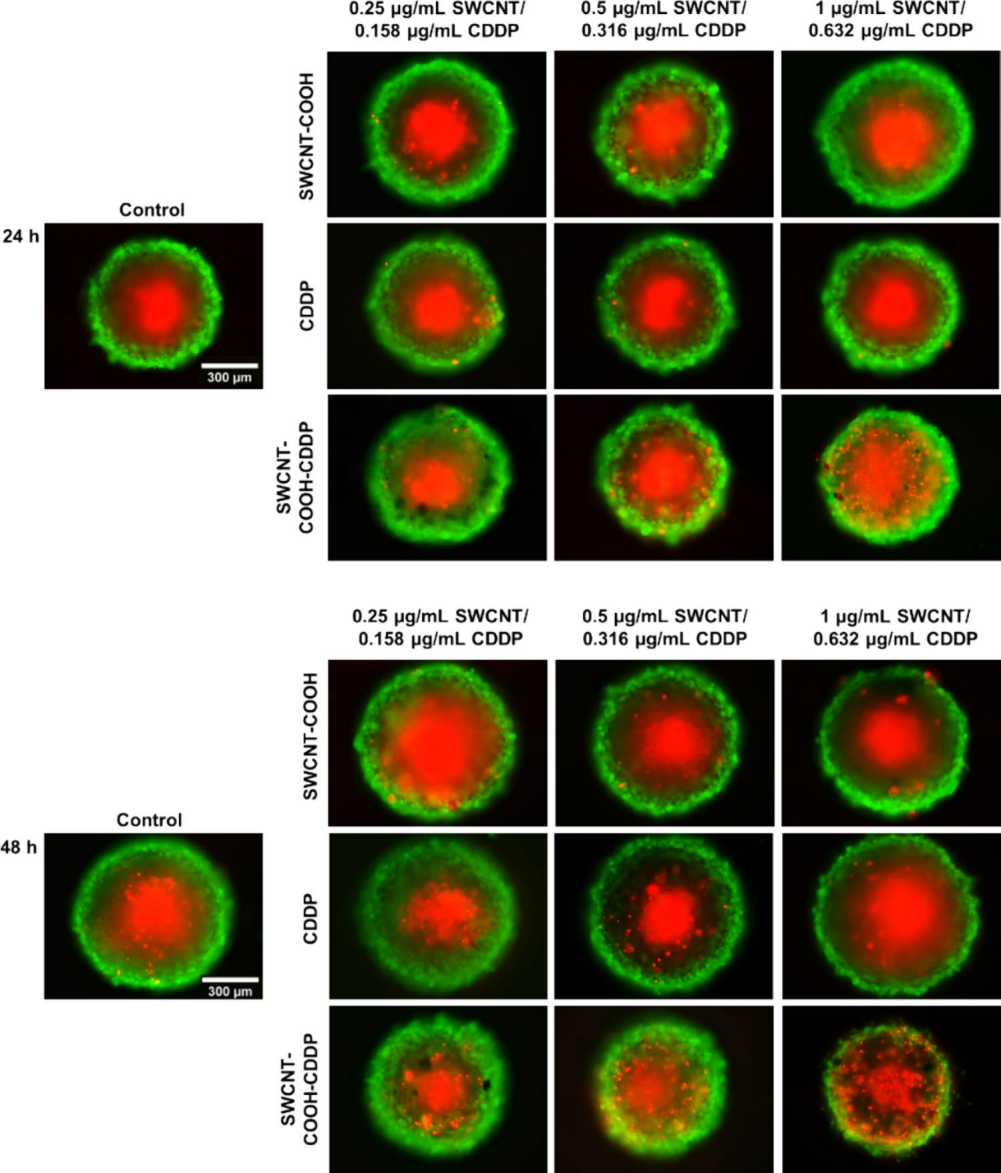
Fluorescence microscopy images presenting the viability of spheroids treated with CDDP (0.158–0.632 μg/mL), SWCNT–COOH (0.25–1 μg/mL) and SWCNT–COOH–CDDP (0.158–0.632 μg/mL) for 24 and 48 h. The concentration of SWCNT–COOH in complex is the same as free SWCNT–COOH. Control represented untreated cultures (0 μg/mL). Green fluorescence corresponds to live cells and red fluorescence indicates dead cells. Scale bar: 300 µm

Due to the significant reduction of cell density in the 2D cultures after exposure to 1.26 μg/mL SWCNT–COOH–CDDP, cell viability by Live/Dead assay was assessed on cells and spheroids treated only with the lowest three doses of complex and free components. The results obtained on 2D cultures were already published and discussed in one of our previous papers (Badea et al. 2022) and highlighted the potential of SWCNT–COOH–CDDP to induce cell death and reduce the density of viable cells. By fluorescent labeling of live and dead cells of 3D-spheroid cultures, it was observed that these present the specific structure of spheroids, a necrotic center surrounded by live cells (Fig. 3). Also, it could be noticed a strong cytotoxic effect of the complex. After 24 h of treatment with 0.632 μg/mL SWCNT–COOH–CDDP, fluorescence images highlighted the presence of a high number of dead cells in the quiescent layer of spheroids. At 48 h, dead cells were observed starting with a dose of 0.158 μg/mL complex and their number increased in a dose-dependent manner. Free components of the complex induced no changes in cell viability of spheroids.

Taking into consideration the results of the Live/Dead assay, two doses of SWCNT–COOH–CDDP, one low (0.316 μg/mL CDDP) and one high (0.632 μg/mL CDDP) were chosen for further biochemical experiments.

**Fig. 4:**
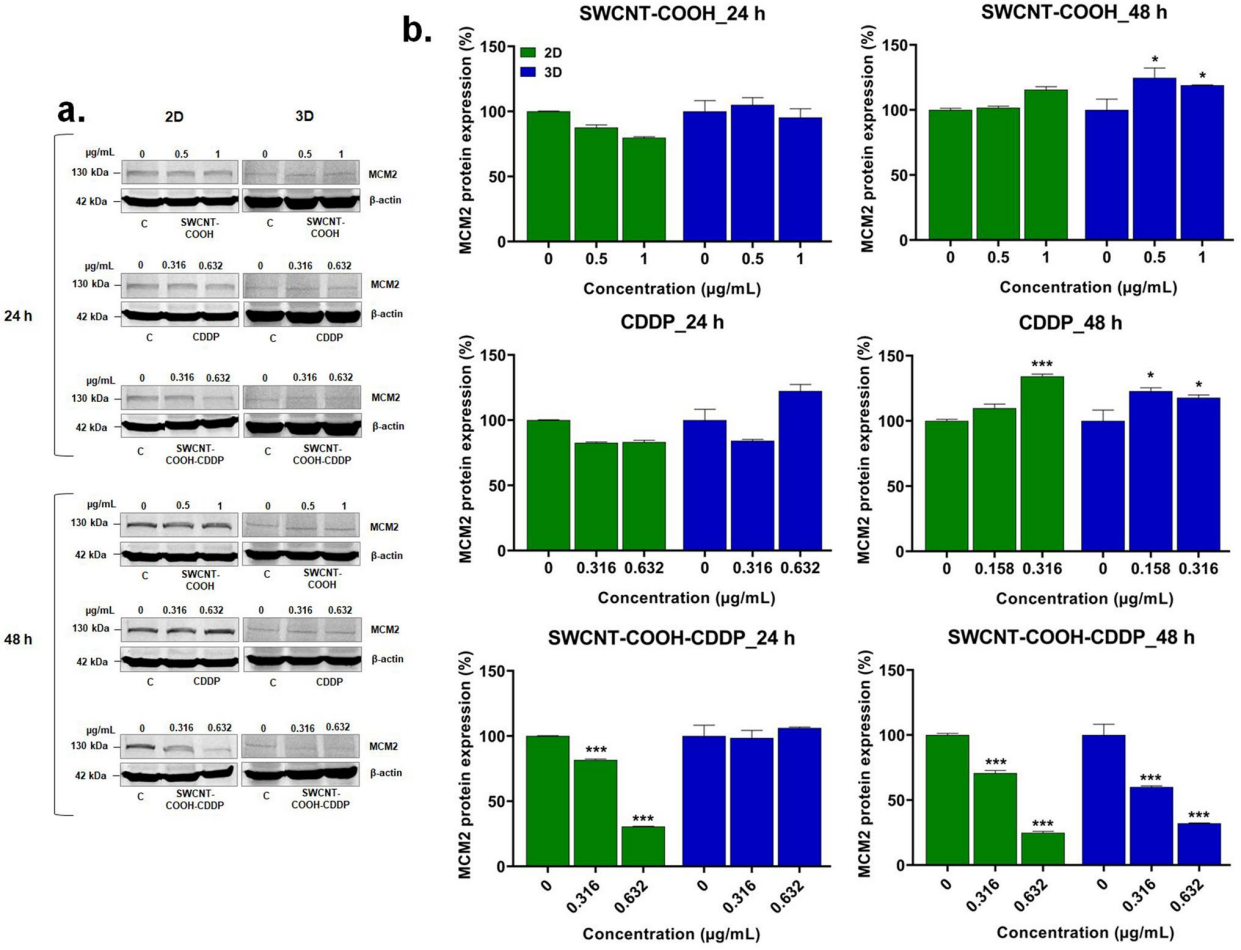
**a** Western blot images representing the expression of MCM2 protein after the exposure of MDA-MB-231 2D and 3D cultures to CDDP (0.316, 0.632 μg/mL), SWCNT–COOH (0.5, 1 μg/mL) and SWCNT–COOH–CDDP (0.316, 0.632 μg/mL) for 24 and 48 h. The concentration of SWCNT–COOH in complex is the same as free SWCNT–COOH. **b** Relative protein expression of MCM2 protein. The bars represent the corresponding quantification of blot images presented in section a of the figure. Untreated cultures (0 μg/mL) were used as control. Results (control vs. sample) were considered significant when *p* < 0.05 (*), *p* < 0.001 (***). Error bars reflect the standard deviation

The capacity of SWCNT–COOH–CDDP complex to inhibit cell proliferation was explored by quantifying the expression of MCM2 protein. In both 2D and 3D cultures, MCM2 expression remained unchanged in the presence of CDDP and SWCNT–COOH after 24 h of treatment. In 2D cultures, the MCM2 expression was down-regulated (by 18.33%, respectively 69.46% compared to control) in a dose-dependent manner after the treatment with SWCNT–COOH–CDDP, while in 3D cultures, no significant modifications compared to control were observed. After 48 h exposure, the expression of MCM2 was up-regulated in 2D cultures in presence of 0.632 μg/mL CDDP, while SWCNT–COOH induced no modifications in protein expression. In the case of MCTSs, both CDDP and SWCNT–COOH samples induced up-regulation of MCM2 expression. Instead, after both intervals of treatment, only SWCNT–COOH–CDDP sample induced the decrease of MCM2 expression in 2D and 3D cultures (after the treatment with 0.316 and 0.632 μg/mL, the expression decreased by 29.9% and 75.16% in 2D cultures and by 39.95% and 67.92% in 3D ones respectively), suggesting the potential of the complex to inhibit cell and spheroid growth (Fig. 4).

### The potential of SWCNT–COOH–CDDP to induce oxidative stress in 2D and spheroid breast cancer cultures

Oxidative stress was assessed by measuring the concentration of reduced glutathione (GSH) as the main non-enzymatic cellular antioxidant and the expression of poly-ubiquitinated Nrf2, a key protein involved in the regulation of cellular antioxidant defense.

**Fig. 5:**
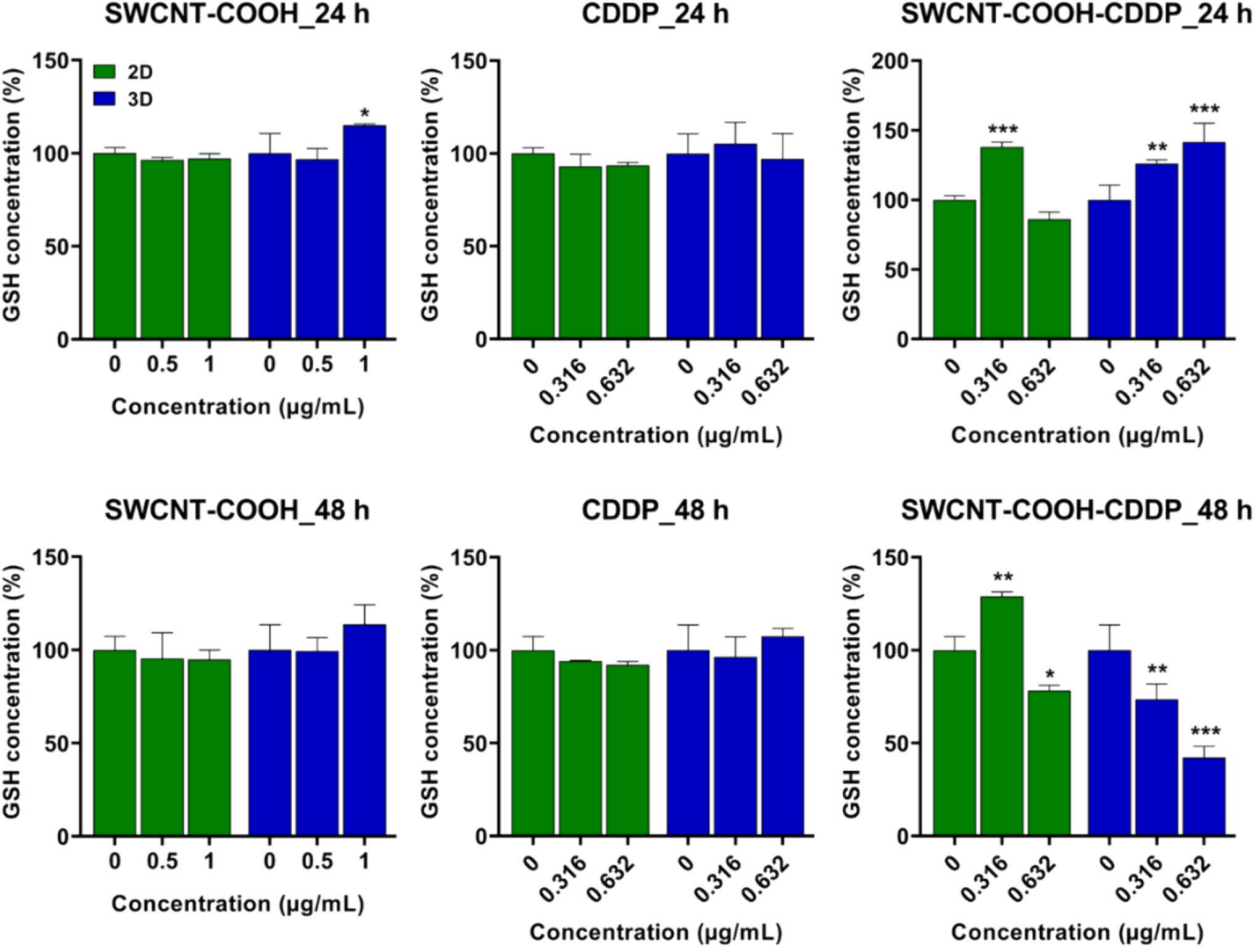
The relative level of reduced glutathione (GSH) in 2D and 3D MDA-MB-231 cultures after treatment with CDDP (0.316, 0.632 μg/mL), SWCNT–COOH (0.5, 1 μg/mL) and SWCNT–COOH–CDDP (0.316, 0.632 μg/mL) for 24 and 48 h. The concentration of SWCNT–COOH in complex is the same as free SWCNT–COOH. Untreated cultures (0 μg/mL) were used as control. Results (control vs. sample) were considered significant when *p* < 0.05 (*), *p* < 0.01 (**), *p* < 0.001 (***). Error bars reflect the standard deviation

In the 2D culture system, GSH level did not change after 24 h exposure to CDDP and SWCNT–COOH, but the exposure of cells to 0.316 μg/mL SWCNT–COOH–CDDP determined a raise of GSH concentration by 38% compared to control. After the same time interval, in 3D cultures, CDDP did not affect this biochemical parameter, whereas 1 μg/mL SWCNT–COOH dose generated a significant increase by 15.1%. A significant increase of GSH concentration by 26.09% and 41.55% was noticed after exposure to 0.316 μg/mL and 0.632 μg/mL SWCNT–COOH–CDDP respectively (Fig. 5).

After 48 h exposure, the GSH concentrations remained at the level of control cells in 2D cultures in the presence of CDDP and SWCNT–COOH, respectively, but the treatment with SWCNT–COOH–CDDP modulated the GSH levels dependent on dose. Thus, the dose of 0.316 μg/mL SWCNT–COOH–CDDP determined an increase of this by 29.08% and that of 0.632 μg/mL SWCNT–COOH–CDDP, a decrease by 21.69%, respectively. In the cells of MCTSs, only SWCNT–COOH–CDDP induced a decrease of GSH concentration in a dose-dependent manner by 26.35% (0.316 μg/mL) and 57.8% (0.632 μg/mL) respectively.

**Fig. 6:**
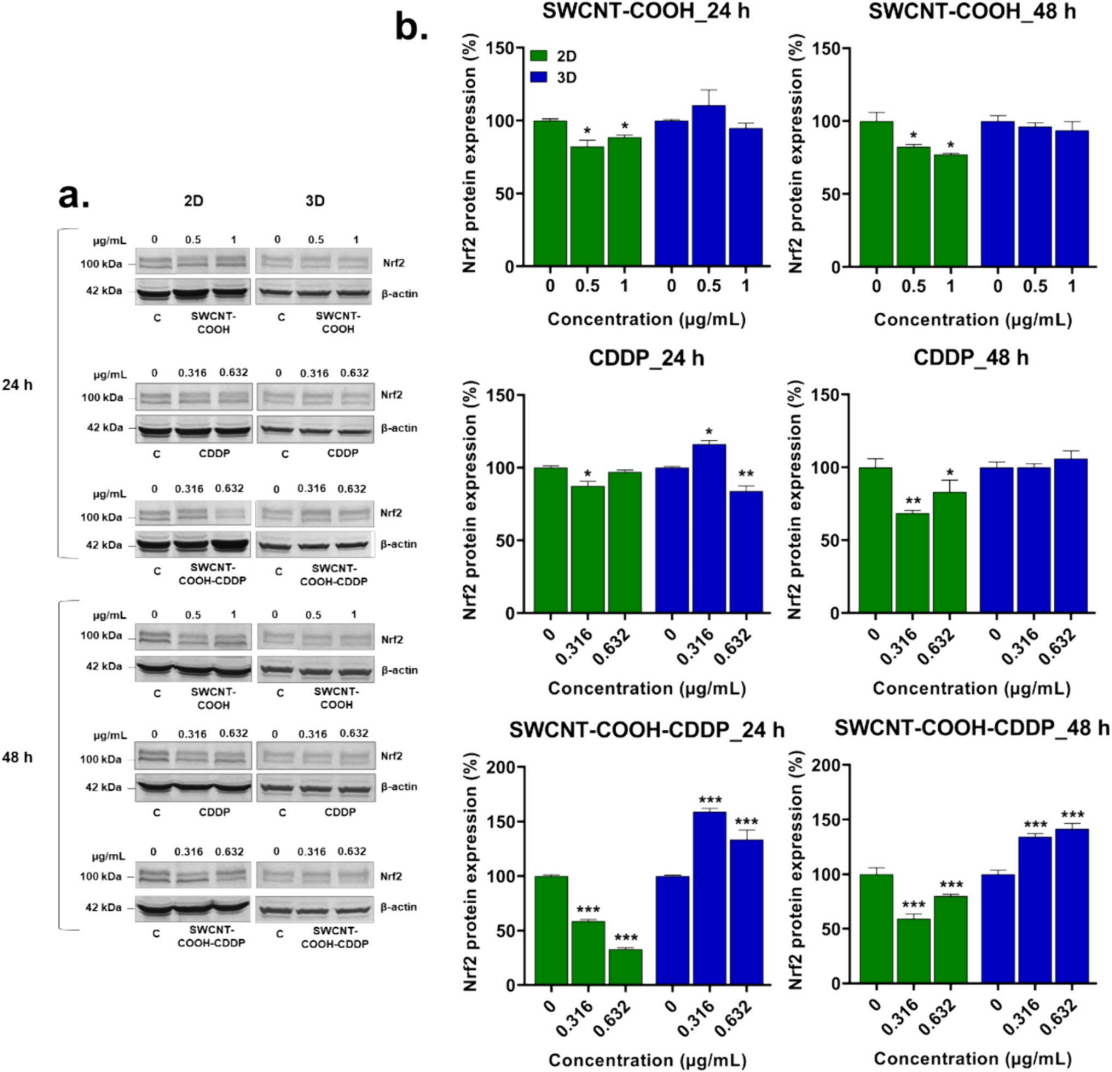
**a** Western blot images representing the expression of poly-ubiquitinated Nrf2 protein after exposure of MDA-MB-231 2D and 3D cultures to CDDP (0.316, 0.632 μg/mL), SWCNT–COOH (0.5, 1 μg/mL) and SWCNT–COOH–CDDP (0.316, 0.632 μg/mL) for 24 and 48 h respectively. The concentration of SWCNT–COOH in complex is the same as free SWCNT–COOH. **b** Relative protein expression of Nrf2 protein. The bars represent the corresponding quantification of blot images presented in section a of the figure. Untreated cultures (0 μg/mL) were used as control. Results (control vs. sample) were considered significant when *p* < 0.05 (*), *p* < 0.01 (**), *p* < 0.001 (***). Error bars reflect the standard deviation

Exposure to SWCNT–COOH–CDDP for 24 and 48 h revealed a different expression of poly-ubiquitinated Nrf2 protein in 2D and 3D cultures (Fig. 6). In 2D cultures, this expression was down-regulated compared to control, while in 3D cultures an up-regulation was registered.

After 24 h, in 2D cultures, the protein expression was down-regulated compared to control after treatment with the low dose of CDDP and both doses of SWCNT–COOH and SWCNT–COOH–CDDP, respectively. The most pronounced down-regulation was registered after the treatment with complex suspension: by 41.36% (0.316 μg/mL) and 67.1% (0.632 μg/mL) respectively. In 3D cultures, the expression was up-regulated after exposure to the low dose of CDDP and both doses of SWCNT–COOH–CDDP and down-regulated after treatment with 0.632 μg/mL CDDP. In the presence of SWCNT–COOH, no significant changes of protein expression were observed in spheroids.

In 2D cultures, the expression of poly-ubiquitinated Nrf2 was down-regulated after 48 h in all experimental conditions. SWCNT–COOH–CDDP induced a decrease by 40.91% (0.316 μg/mL) and 20.24% (0.632 μg/mL) respectively, whereas free CDDP down-regulated the expression only by 31.41% (0.316 μg/mL) and 16.71% (0.632 μg/mL) respectively. In 3D cultures, the expression of poly-ubiquitinated Nrf2 protein remained unchanged after exposure to free components. Instead, SWCNT–COOH–CDDP induced an up-regulation of protein expression by 34.3% (low dose) and by 41.44% (high dose).

### Cell death pathways activated in 2D and 3D cultures after the exposure to SWCNT–COOH–CDDP complex

After exposure of MDA-MB-231 cells and spheroids to SWCNT–COOH–CDDP, apoptosis was evaluated, as the main programmed cell death mechanism induced by CDDP (Dasari and Tchounwou 2014). Activation of apoptosis was explored by the analyses of mitochondrial membrane potential (MMP), as an indicator of mitochondrial function, and protein expression of caspase-3, caspase-8, caspase-9, caspase-4.

**Fig. 7:**
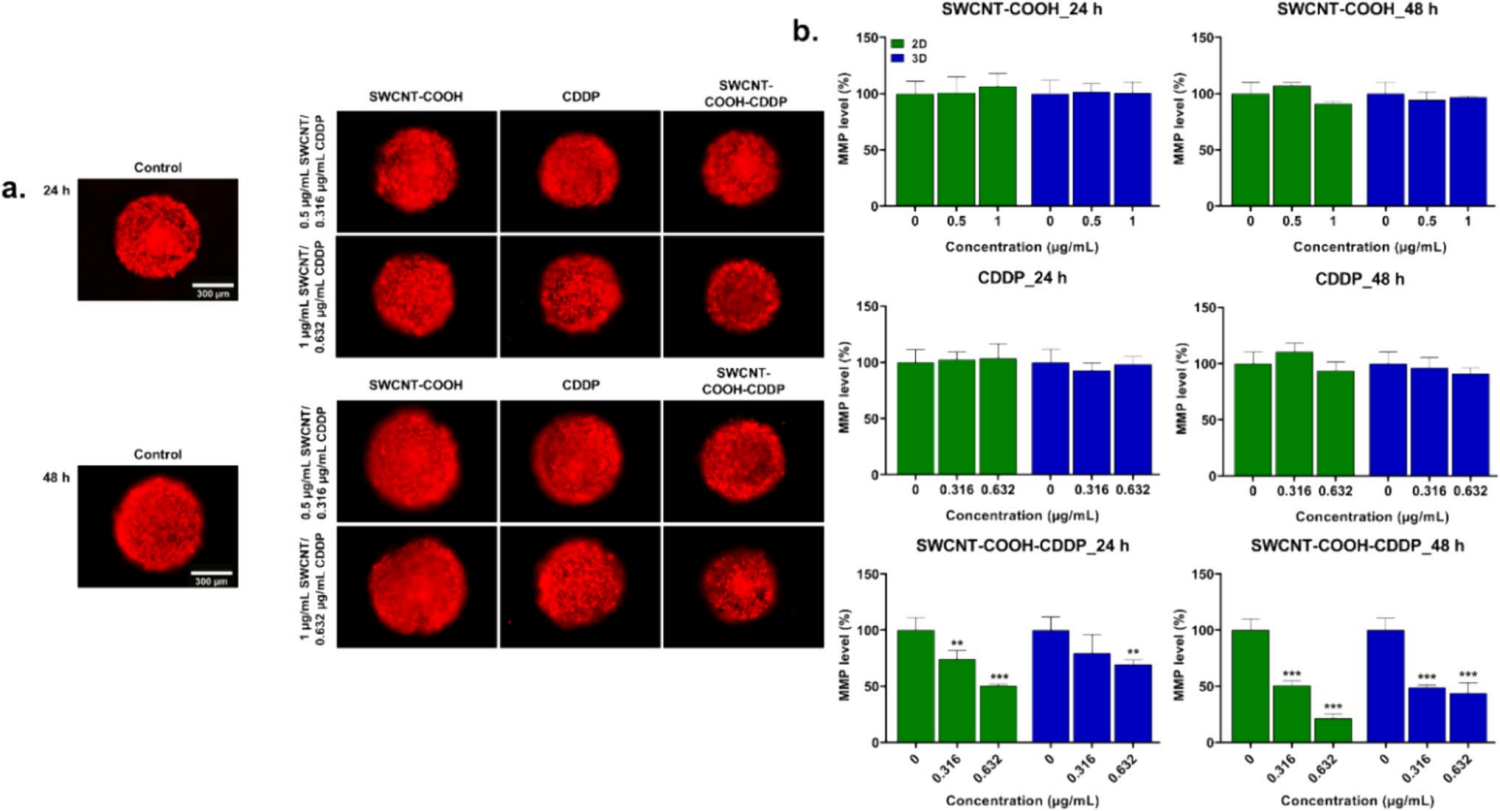
**a** Microscopy images presenting the fluorescence corresponding to mitochondrial membrane potential of MDA-MB-231 spheroids treated with CDDP (0.316, 0.632 μg/mL), SWCNT–COOH (0.5, 1 μg/mL) and SWCNT–COOH–CDDP (0.316, 0.632 μg/mL) for 24 and 48 h. The concentration of SWCNT–COOH in complex is the same as free SWCNT–COOH. **b** Relative mitochondrial membrane potential of 2D and 3D cultures after treatment. In the case of 3D-spheroid cultures, the bars represent the corresponding fluorescence quantification of the images presented in section a of the figure. Untreated cultures (0 μg/mL) were used as control. Results (control vs. sample) were considered significant when *p* < 0.01 (**), *p* < 0.001 (***). Error bars reflect the standard deviation

Free components caused no significant modifications of MMP in both experimental culture models. In contrast, the complex induced a decrease of MMP level in a time- and dose-dependent manner in MDA-MB-231 cells, being more pronounced in the 2D cultures compared to 3D ones. In 3D-spheroid cultures, MMP level decreased significantly after 24 h treatment with the dose of 0.632 μg/mL (by 30.47%) and after 48 h incubation with both doses by 50.98% and 56.02% respectively. In the cells of 2D cultures, MMP decreased significantly after 24 h by 25.87% and 49.42% after treatment with 0.316 μg/mL respectively 0.632 μg/mL. A higher reduction by 49.15% and by 78.41% respectively was noticed after 48 h post-treatment (Fig. 7).

**Fig. 8:**
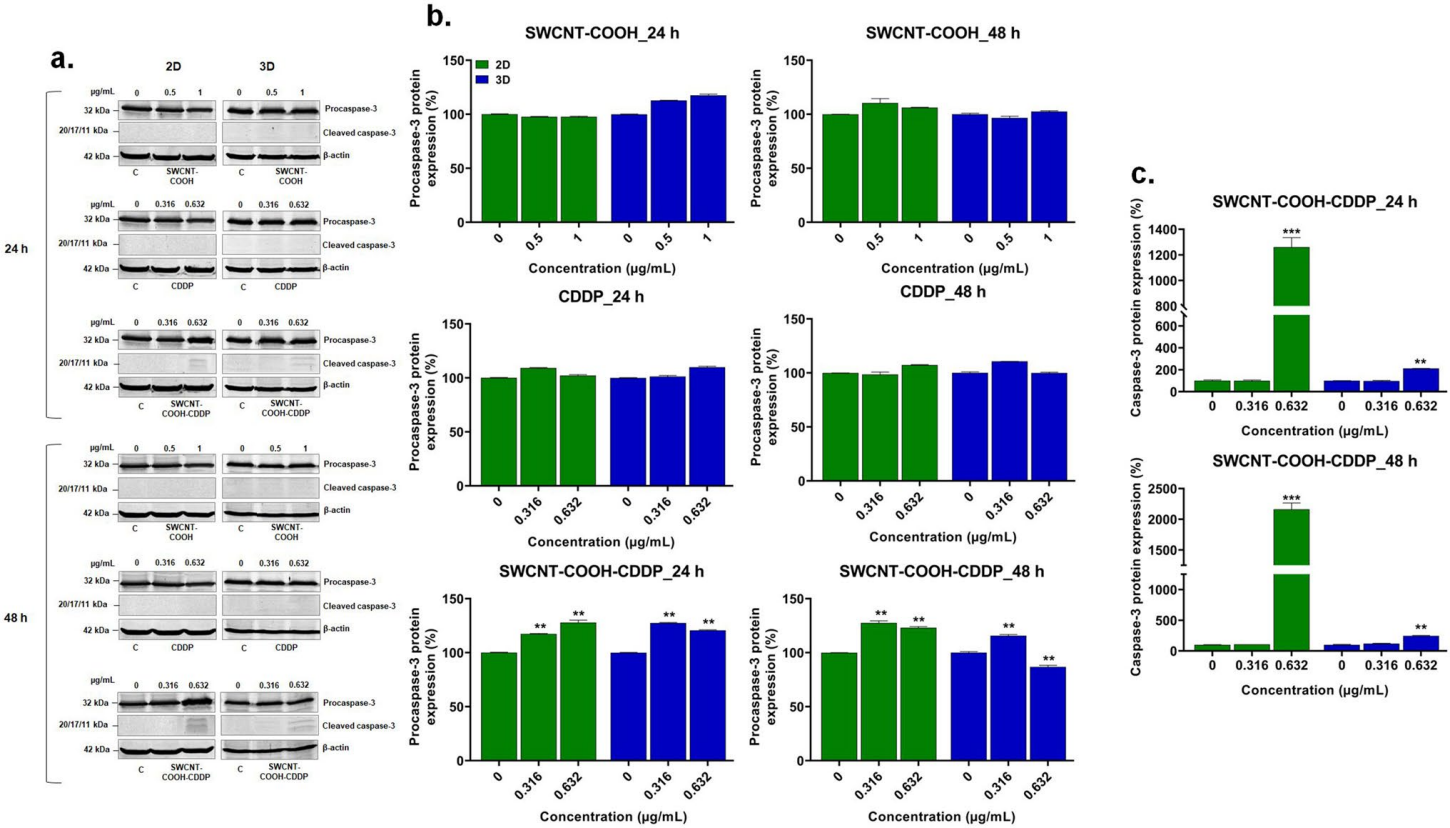
**a** Western blot images representing the expression of procaspase-3 and caspase-3 proteins after exposure of MDA-MB-231 2D and 3D cultures to CDDP (0.316, 0.632 μg/mL), SWCNT–COOH (0.5, 1 μg/mL) and SWCNT–COOH–CDDP (0.316, 0.632 μg/mL) for 24 and 48 h. The concentration of SWCNT–COOH in complex is the same as free SWCNT–COOH. Relative protein expression of procaspase-3 (**b**) and capsase-3 (**c**) proteins. The bars represent the corresponding quantification of blot images presented in section a of the figure. Untreated cultures (0 μg/mL) were used as control. Results (control vs. sample) were considered significant when *p* < 0.01 (**), *p* < 0.001 (***). Error bars reflect the standard deviation

The expression of procaspase-3 and caspase-3 proteins was not significantly modified by the treatment with CDDP and SWCNT–COOH in breast cancer 2D and 3D-spheroid cultures (Fig. 8).

The results were different after the exposure to SWCNT–COOH–CDDP. The expression of procasapse-3 increased compared to control in both 2D and 3D cultures in all experimental conditions, except for the 3D culture exposed to 0.632 μg/mL, after 48 h, when it was down-regulated compared to untreated spheroids. Moreover, the expression of caspase-3 was up-regulated in a time-dependent manner compared to control after the incubation with 0.632 μg/mL SWCNT–COOH–CDDP in 2D cultures. The level of caspase-3 expression in 2D cultures exceeded the one found in 3D cultures by 1918.28% after 48 h. No activation of caspase-3 was found after treatment with the lower dose of complex in the cells of 2D and 3D systems. Taking into account these results, the protein expression of several upstream caspases involved in the activation of caspase-3 have been investigated.

Extrinsic pathway of apoptosis was explored by analyses of the expression of procaspase-8 and caspase-8 after exposure of the cells of 2D and 3D cultures to the high dose of the complex and free components.

After 24 h exposure, expression of procaspase-8 in the cells of 2D cultures was unchanged after treatment with the complex and free components. The same results were obtained also for 3D cultures at 24 h, except for the sample of SWCNT–COOH–CDDP (dose of 0.632 μg/mL) when an increase by 24.03% of procaspase-8 expression was noticed. At the same interval, SWCNT–COOH–CDDP induced an up-regulation by 59.27% of caspase-8 expression in the cells in 2D systems. No significant changes of caspase-8 expression were observed after 24 h in any other experimental conditions.

**Fig. 9:**
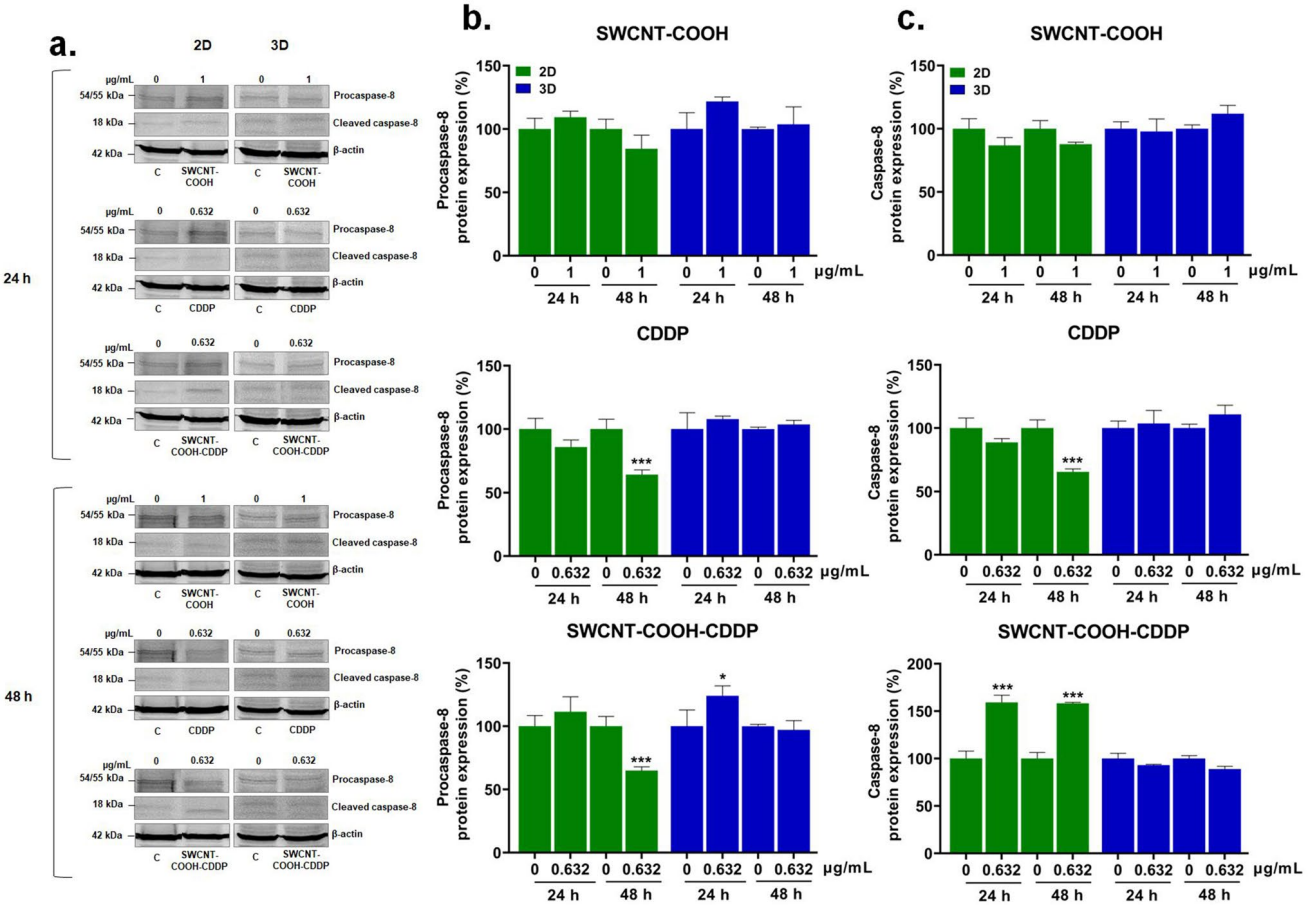
**a** Western blot images representing the expression of procaspase-8 and caspase-8 proteins after exposure of MDA-MB-231 2D and 3D cultures to CDDP (0.632 μg/mL), SWCNT–COOH (1 μg/mL) and SWCNT–COOH–CDDP (0.632 μg/mL) for 24 and 48 h respectively. The concentration of SWCNT–COOH in complex is the same as free SWCNT–COOH. Relative protein expression of procaspase-8 (**b**) and capsase-8 (**c**) proteins. The bars represent the corresponding quantification of blot images presented in section a of the figure. Untreated cultures (0 μg/mL) were used as control. Results (control vs. sample) were considered significant when *p* < 0.05 (*), *p* < 0.001 (***). Error bars reflect the standard deviation

The expression of procaspase-8 in 2D cultures was down-regulated by 37.73% and 35.11% after 48 h treatment with CDDP and SWCNT–COOH–CDDP respectively. SWCNT–COOH did not affect the expression of this protein in 2D systems. In the case of 3D-spheroid cultures, no significant changes were noticed. Moreover, after this time interval, caspase-8 expression level decreased by 34.4% and increased by 58.24% after exposure to CDDP and SWCNT–COOH–CDDP respectively, in the cells in 2D cultures. No significant modulation of caspase-8 expression was observed after the treatment of 3D-spheroid cultures with the complex and free components (Fig. 9).

**Fig. 10:**
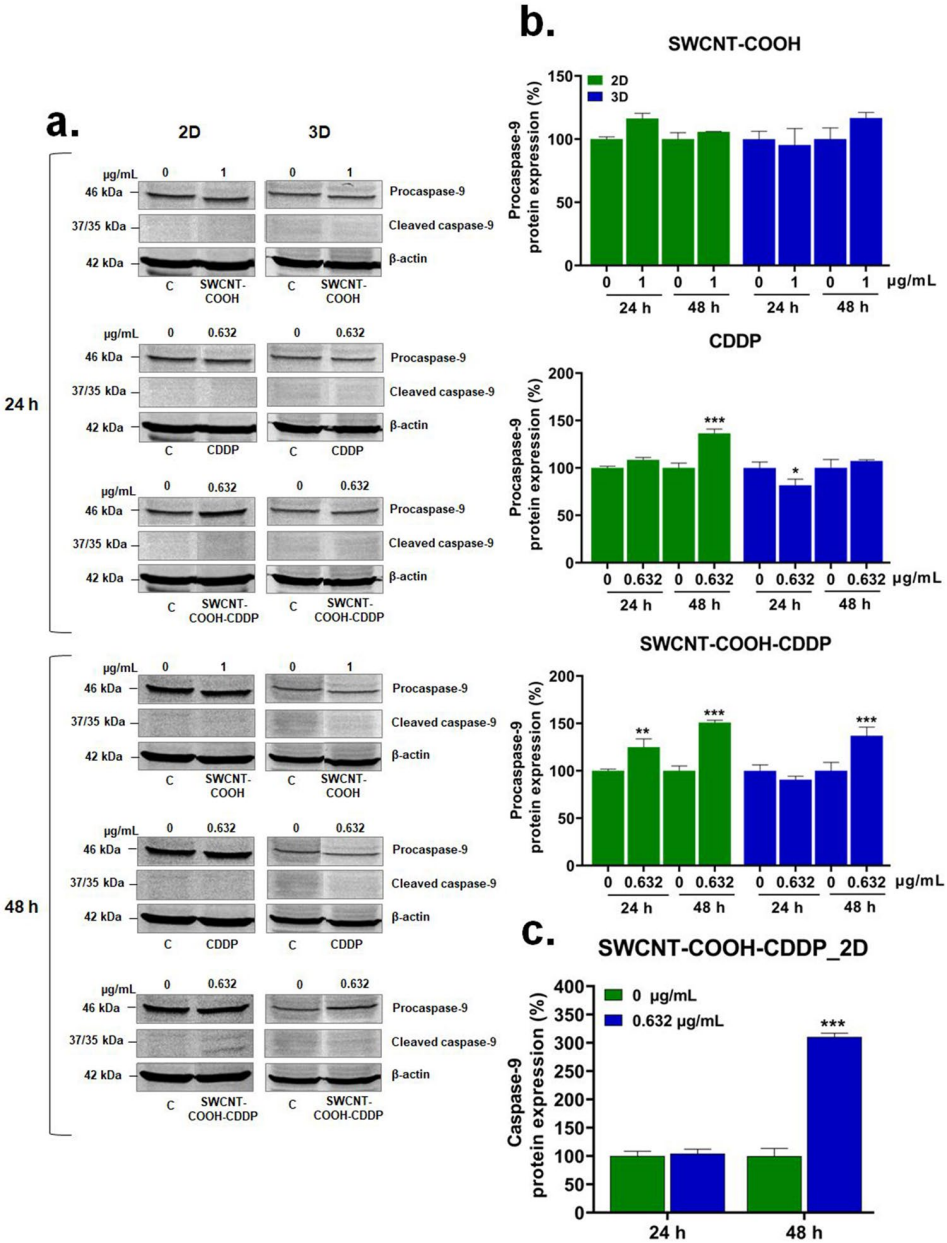
**a** Western blot images representing the expression of procaspase-9 and caspase-9 proteins after exposure of MDA-MB-231 2D and 3D cultures to CDDP (0.632 μg/mL), SWCNT–COOH (1 μg/mL) and SWCNT–COOH–CDDP (0.632 μg/mL) for 24 and 48 h. The concentration of SWCNT–COOH in complex is the same as free SWCNT–COOH. Relative protein expression of procaspase-9 (**b**) and capsase-9 (**c**) proteins. The bars represent the corresponding quantification of blot images presented in section a of the figure. Untreated cultures (0 μg/mL) were used as control. Results (control vs. sample) were considered significant when* p* < 0.01 (**), *p* < 0.001 (***). Error bars reflect the standard deviation

Activation of intrinsic pathway of apoptosis was investigated by quantifying the expression of caspase-9 and procaspase-9 (Fig. 10). As it can be seen from Western blot images, caspase-9 was activated only in 2D cultures exposed to 0.632 μg/mL SWCNT–COOH–CDDP for 48 h. The same sample induced an elevation of procaspase-9 protein expression in 2D cultures in a time-dependent manner (by 24.94% and 50.93% respectively), and in spheroids after 48 h (by 36.97%). Free CDDP induced an increase of procaspase-9 expression in 2D cultures after 48 h and the decrease in 3D-spheroid cultures after 24 h. After treatment of MDA-MB-231 cells in 2D and 3D systems with SWCNT–COOH, the expression of procaspase-9 remained similar with the one of control cells.

**Fig. 11:**
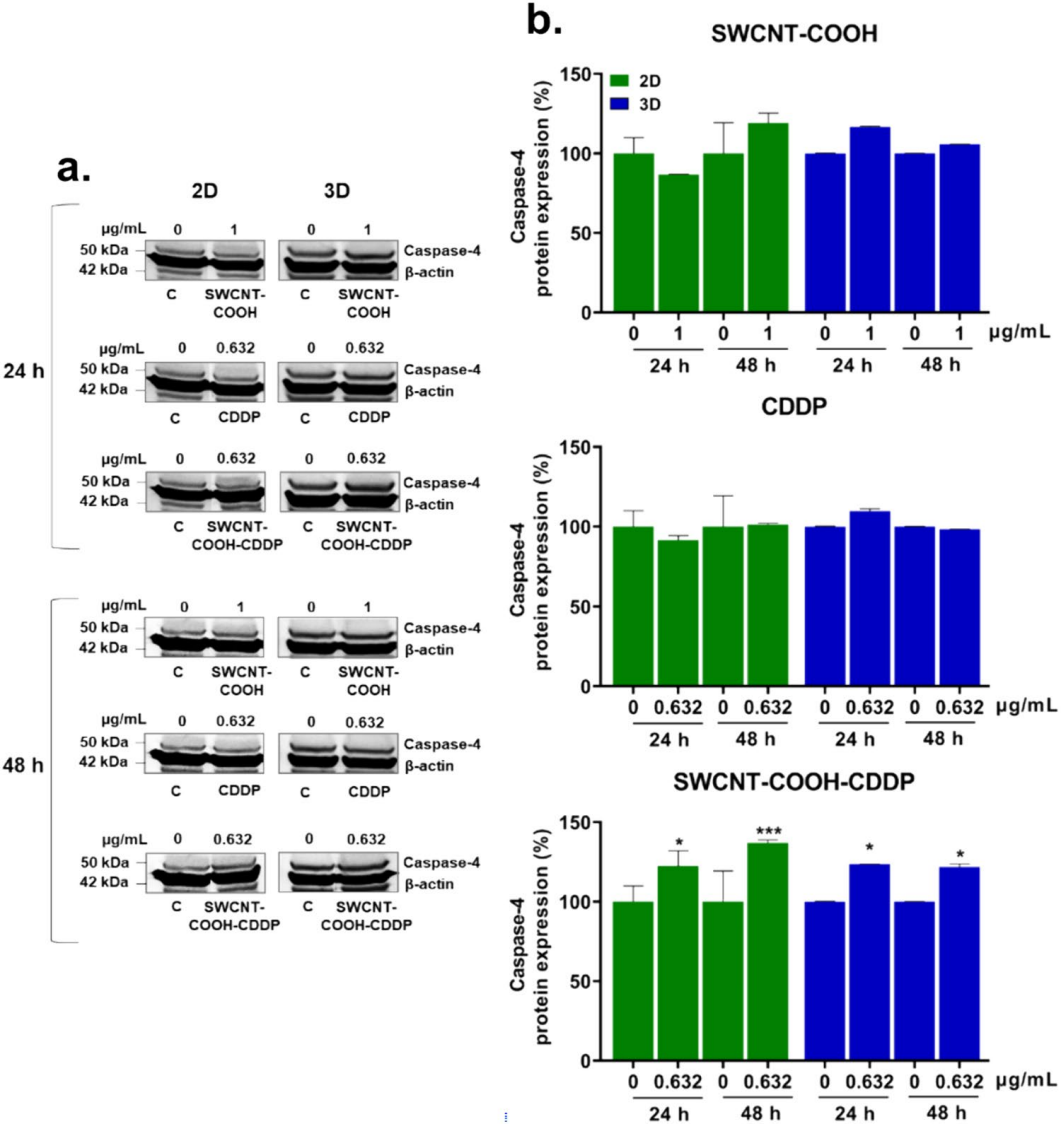
**a** Western blot images representing the expression of caspase-4 protein after exposure of MDA-MB-231 2D and 3D cultures to CDDP (0.632 μg/mL), SWCNT–COOH (1 μg/mL) and SWCNT–COOH–CDDP (0.632 μg/mL) for 24 and 48 h. The concentration of SWCNT–COOH in complex is the same as free SWCNT–COOH. **b** Relative protein expression of capsase-4 protein. The bars represent the corresponding quantification of blot images presented in section a of the figure. Untreated cultures (0 μg/mL) were used as control. Results (control vs. sample) were considered significant when *p* < 0.05 (*), *p* < 0.001 (***). Error bars reflect the standard deviation

Furthermore, the expression of caspase-4 protein was investigated as a marker of endoplasmic reticulum stress-induced apoptosis. Quantification of Western blot images revealed no activation of caspase-4 after treatment of 2D and 3D-spheroid cultures with CDDP and SWCNT–COOH for 24 and 48 h. Instead, SWCNT–COOH–CDDP complex induced the overexpression of caspase-4 compared to control in both breast cancer 2D and 3D cultures. Thus, the expression increased by 22.56% and 36.97% in breast cancer cells in 2D system and by 23.57% and 21.72% in spheroids, after 24 and 48 h, respectively (Fig. 11).

### The potential of SWCNT–COOH–CDDP to inhibit the metastasis of 2D and 3D breast cancer cultures

The potential of breast cancer 2D and 3D-spheroid cultures to invade the surrounding matrix and develop metastasis was assessed after treatment with the low and high doses of the complex and free components for 24 and 48 h. After 24 h of treatment, CDDP and SWCNT–COOH–CDDP samples caused an inhibition of invasion potential of 2D cultures, while in 3D cultures only SWCNT–COOH–CDDP reduced the invasion potential by 26.42% (dose of 0.316 μg/mL) and by 63% (dose of 0.632 μg/mL). SWCNT–COOH induced no effects after 24 h in 2D and 3D cultures.

**Fig. 12:**
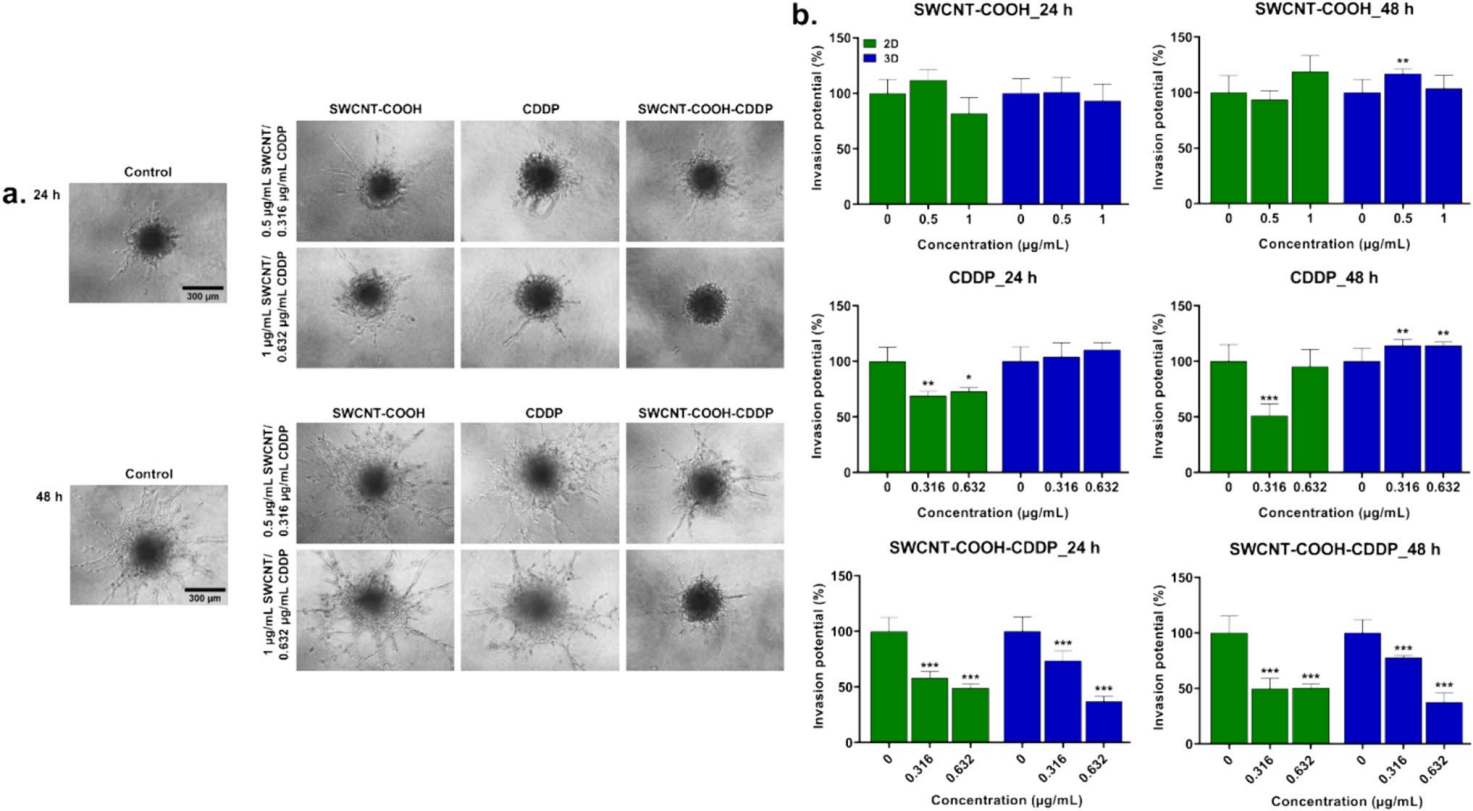
**a** Microscopy images presenting the invasion rate into surrounding matrix of MDA-MB-231 spheroids treated with CDDP (0.316, 0.632 μg/mL), SWCNT–COOH (0.5, 1 μg/mL) and SWCNT–COOH–CDDP (0.316, 0.632 μg/mL) for 24 and 48 h. The concentration of SWCNT–COOH in complex is the same as free SWCNT–COOH. **b** Relative invasion potential of 2D and 3D cultures after treatment. In the case of 3D-spheroid cultures, the bars represent the corresponding invasion of cells into surrounding matrix presented in the section a images of the figure. Untreated cultures (0 μg/mL) were used as control. Results (control vs. sample) were considered significant when *p* < 0.05 (*), *p* < 0.01 (**), *p* < 0.001 (***). Error bars reflect the standard deviation

After 48 h exposure, the low dose of CDDP decreased the invasion potential of 2D cultures (by 48.84%). SWCNT–COOH had no influence on the potential of MDA-MB-231 cells cultured in a 2D system to cross the polycarbonate membrane. SWCNT–COOH–CDDP inhibited the invasion of MDA-MB-231 cells by 50.27% and 49.37% after 48 h treatment with 0.316 μg/mL and 0.632 μg/mL respectively (Fig. 12). In 3D cultures, both doses of CDDP induced a stimulation of invasion capacity (by 14.07% and 14.1% respectively). The degree of invasiveness of cells cultures in 3D systems was amplified by the treatment with 0.5 μg/mL SWCNT–COOH (by 17.12%), and inhibited by 0.316 μg/mL and 0.632 μg/mL SWCNT–COOH–CDDP with 22.31% and 62.47% respectively, compared to control.

## Discussion

The bi-dimensional (2D) and tri-dimensional (3D) triple-negative breast cancer cultures were used for assessment of the activity of free cisplatin and complexed with carbon nanotubes. Single and multiple multicellular tumor spheroid (MCTSs) cultures generated by liquid-overlay technique in medium with 2.5% Matrigel served as 3D cultures. The use of both types of spheroid cultures was motivated by the high similarity of these, as it was proved in one of our previous studies. Single and multiple MDA-MB-231 3D cultures were similar in proliferative capacity (PCNA expression) and development of hypoxia process (Nrf2 expression and GSH levels), which can indicate a good reproducibility of solid tumors characteristics and validate the extrapolation of the results from single to multiple spheroid cultures (Badea et al. 2019).

Although traditional 2D cell cultures are characterized by a poor recapitulation of in vivo complexity due to the lack of extracellular matrix and changes in cell morphology and polarity, they are still the most used and accessible systems in drug screening. These limitations of 2D cultures induce a susceptibility of cells to drug action and lead to an incorrect estimation of their efficiency (Kapałczyńska et al. 2018; Foglizzo et al. 2022). In contrast, 3D cell cultures are more predictive and relevant models and reproduce better the in vivo environment, offering a more accurate representation of physiological response to drugs (Tang et al. 2023).

Various studies reported a higher activity of many chemotherapeutic drugs on 2D cell cultures, compared to 3D (Melissaridou et al. 2019; Muguruma et al. 2020). Consistent with these studies, we observed more pronounced cytotoxicity of SWCNT–COOH–CDDP in 2D cultures compared to spheroids, indicated by the higher release of lactate dehydrogenase (LDH) in the culture medium. In 2D cell cultures, the release of LDH started to increase after 24 h (dose of 0.632 μg/mL), while in the medium of spheroids, its activity was raised only after 48 h of treatment with the highest dose (1.26 μg/mL). This could suggest the high susceptibility of MDA-MB-231 2D cultures to CDDP action compared to spheroids. Permeabilization of the plasma membrane is a key feature of cells undergoing apoptosis, necrosis, and other forms of cellular damage (Kumar et al. 2018). In correlation with this, analysis of cell morphology revealed that MDA-MB-231 cells cultured in 2D systems were characterized by morphological features specific to apoptosis after exposure to SWCNT–COOH–CDDP, starting with the dose of 0.632 μg/mL: cell shrinkage, losing of cell volume and cell–cell adhesions, vacuoles containing cytoplasm, cell fragmentation (Bortner and Cidlowski 1998; Elmore 2007; Nikoletopoulou et al. 2013). Instead, in 3D cultures, due to the specific growth of spheroids, analysis of morphology indicated only the reduction of spheroids’ dimensions and the perturbation of proliferative layer integrity after the treatment with SWCNT–COOH–CDDP complex, which could not be associated with a type of cell death. However, the reduction of spheroids’ dimensions was confirmed by the down-regulation of MCM2 protein expression by SWCNT–COOH–CDDP after 48 h, which highlighted the inhibition of proliferative rate. Mini-chromosome maintenance 2 (MCM2), a protein involved in the initiation of DNA replication, is expressed in actively proliferating cells and non-cycling cells with proliferative potential, being considered a proliferation marker (Yousef et al. 2017). Inhibition of proliferative potential by the complex sample was induced also in 2D cultures after both intervals, as indicated by the decrease of MCM2. Furthermore, the results revealed a time- and dose-dependent inhibition of proliferative rate, with a higher susceptibility of 2D cultures compared to 3D, excepting the dose of 0.316 μg/mL SWCNT–COOH–CDDP after 48 h when the level of MCM2 in breast spheroids was lower than in 2D cultures. This can be explained by the high amount of nutrients, growth factors, and oxygen available in 2D cultures, which can support the proliferation of cells. Also, this can be associated with the predominance of proliferative cells in 2D cultures compared to spheroids which are formed by cells of different stages, including necrotic cells and it was demonstrated also on prostate tumor cell lines exposed to paclitaxel and docetaxel (Souza et al. 2018). However, the effects reversed after the treatment with 0.632 μg/mL SWCNT–COOH–CDDP for 48 h may be because of the stronger effect of this dose. In contrast with the decrease of MCM2 expression induced by SWCNT–COOH–CDDP sample, the increase of this protein expression by CDDP and SWCNT–COOH could suggest a stimulation of the proliferative potential of cells. In accordance with our results, similar studies demonstrated the efficiency of chemotherapeutics in inhibiting the growth rate of 2D and 3D-spheroid cultures. Thus, Uematsu and collaborators (2018) proved less sensitivity of breast cancer MCF-7 spheroids to the anti-proliferative efficacies of anti-cancer agents than the monolayer cultures (Uematsu et al. 2018).

The reduction of spheroids growth and the increased production of LDH registered after complex exposure were confirmed also by the results obtained by Live/Dead assay, which suggest the potential of SWCNT–COOH–CDDP to induce cell death. A dose- and time-dependent increase in dead cell number was highlighted in breast cancer cells distributed predominantly in the quiescent zone of spheroids, highlighting its sensitivity and the role of the proliferative layer in maintaining spheroid viability. Similar results obtained for 2D cultures, which revealed strong cytotoxicity of the complex, were presented and discussed in our previous paper (Badea et al. 2022).

Cisplatin (CDDP) mainly acts in the cells by covalent binding of platinum to guanine and adenine bases from the DNA structure. Besides this, CDDP can induce cytotoxicity by producing oxidative stress (Yu et al. 2018). In this study, we evaluated the occurrence of oxidative stress by determination of reduced glutathione (GSH) level and quantification of poly-ubiquitinated Nrf2 protein expression. Oxidative stress is defined as the intracellular condition that results when the production of oxidant molecules (known as reactive oxygen species) exceeds the antioxidant capacity of cells (Pizzino et al. 2017). GSH is the most abundant antioxidant in the cells with important roles in buffering oxidative stress and cancer cells' response to treatment (Asantewaa and Harris 2021). The 2D and 3D breast cancer cultures treated with SWCNT–COOH–CDDP produced the most significant outcomes for GSH level, with both stimulation and inhibition being noted. Elevation of GSH found following exposure of 2D cultures (0.316 μg/mL for 24 and 48 h) and 3D cultures (both doses after 24 h) to the complex may thus point to a potential antioxidant cell capacity that alleviates oxidative damage. In contrast, diminished levels of GSH were registered in the case of 2D (dose of 0.632 μg/mL) and 3D (both doses) after 48 h of treatment, which may indicate a cellular susceptibility toward oxidative stress after a longer exposure of cells to complex (Gawryluk et al. 2011). According to these findings, the oxidative stress could be more pronounced in 3D compared to 2D cultures. This can be motivated by the presence of the hypoxic region in the spheroid center. A hypoxic medium is associated with increased levels of reactive oxygen species and induction of oxidative stress (D’Aiuto et al. 2022) and, as a consequence, hypoxic cells could be more sensitive to oxidant conditions induced by extrinsic stimuli. Similar results were obtained also when perfused 3D cell cultures and traditional 2D cultures were exposed to CDDP (Liu et al. 2018) and after chronic stress exposure of 2D and 3D in vitro models of human trabecular meshwork cells (Vernazza et al. 2019). Additionally, the activation of antioxidant mechanisms and the exocytosis of CNT from MDA-MB-231 spheroids can account for the increase in GSH level following the treatment with 1 μg/mL SWCNT–COOH for 24 h and the subsequent return to control level at 48 h.

The nuclear factor erythroid 2-related factor 2, also known as Nrf2, is an essential regulator of the GSH synthesis and utilization enzymes and is involved in the cellular defense against oxidative stress. Under non-stress conditions, this protein is subjected to ubiquitination and proteasomal degradation as a result of its association with Kelch-like ECH-associated protein (Keap1). Under oxidative stress, Nrf2 is prevented from being ubiquitinated, which causes its stabilization, accumulation, and nuclear translocation. Once it reaches the nucleus, regulates the expression of the genes for the enzymes involved in detoxification (Ngo and Duennwald 2022).

Taking into account Nrf2 signaling, we evaluated the expression of poli-ubiquitinated Nrf2 (~ 100 kDa), and important differences between MDA-MB-231 2D and 3D spheroid cultures following the exposure to the complex were noted. In 2D cultures, SWCNT–COOH–CDDP caused the down-regulation of Nrf2 expression which could indicate the translocation of protein into nucleus, while in 3D cultures, the results were reversed and the protein expression increased indicating that the protein has accumulated in cytoplasm. The change of Nrf2 expression in the presence of CDDP and SWCNT–COOH can be similarly explained. However, taking into consideration the GSH level registered in 2D and 3D cultures exposed to the complex sample, it could be postulated that oxidative stress level registered in the 3D system did not reach the required threshold to facilitate the translocation of Nrf2 protein into the nucleus of cells.

CDDP forms inter- and intra-strand crosslinked DNA adducts which initiate a cascade of events that culminates with apoptosis (Tanida et al. 2012). Additionally, alterations in biomolecules brought on by reactive oxygen species action might cause oxidative stress, which also leads to apoptosis (Redza-Dutordoir and Averill-Bates 2016). Apoptosis is the programmed cell death mediated by proteolytic enzymes, known as caspases. Caspases are cysteine proteases synthesized as inactive precursors or zymogens (procaspases) and are catalytically activated in the apoptotic proteolytic cascade. These are divided into initiator caspases (caspase-2, -8, -9, and -10) which initiate the apoptosis signal, and executioner caspases (caspase-3, -6, and -7) which induce the proteolysis of target molecules and lead to apoptosis (Alberts et al. 2002; Shi 2004). Apoptosis has been described as a complex mechanism with the involvement of two apoptotic pathways: extrinsic, based on transmembrane receptor-mediated interactions, and intrinsic or mitochondrial pathway (Elmore 2007). The most important executioner caspase is caspase-3, which can be activated by both intrinsic and extrinsic pathways and cleaves proteins with crucial roles in cell survival (Seervi and Xue 2015). After exposure of breast cancer 2D and 3D cultures to complex and free components, only a dose of 0.632 μg/mL SWCNT–COOH–CDDP activated caspase-3 in both 2D and 3D cultures after 24 and 48 h, respectively, demonstrating the activation of the apoptotic cell death pathway. These results could be positively correlated with the ones obtained by Live/Dead and LDH assays. The absence of caspase-3 activation in the presence of free CDDP highlights that cells were not susceptible to apoptosis. Moreover, the higher expression of caspase-3 in 2D cultures compared to 3D could be linked with the high susceptibility of MDA-MB-231 cells to the action of CDDP released from the complex and can be explained by the high compaction of 3D cultures which might play a role in the cell protection against apoptosis. On top of that, in 2D cultures, apoptosis was induced concomitantly with the inhibition of the PI3K/Akt signaling pathway, as it was presented in our previous study (Badea et al. 2022). The importance of 3D cultures compaction was underlined also by Liu and collaborators (2018) who found that the cells grown in 3D cultures were more resistant to CDDP drug compared to those grown in 2D cultures (Liu et al. 2018). Similar results were also noticed after paclitaxel exposure in breast cancer cultures: caspase-3 expression in the BT-474 cell line was lower in 3D culture than in 2D culture, indicating an anti-apoptotic environment in the 3D cultures (Imamura et al. 2015). Simultaneously, SWCNT–COOH–CDDP induced the overexpression of procaspase-3 in all experimental conditions, except the 3D cultures treated with 0.632 μg/mL for 48 h when the expression was inhibited. Previously, it was shown that overexpression of procaspase-3 is correlated with the sensitization of ovarian cancer cells to proteasome inhibitors (Tenev et al. 2001).

The activation of the extrinsic pathway of apoptosis was investigated using quantification of caspase-8 protein expression. Caspase-8 can be activated by proteolytic cleavage through dimerization and autoactivation. Procaspase-8's catalytic activity is activated by dimerization, allowing it to release caspase-8 into the cytosol where it cleaves caspase-3 and causes apoptosis (Orning and Lien 2021). According to our data, caspase-8 activation was only observed in 2D cultures after the treatment with complex for both intervals, revealing the involvement of extrinsic pathway in apoptosis signaling. In contrast with SWCNT–COOH–CDDP, free CDDP induced the inhibition of caspase-8 expression in 2D cultures, after 48 h. Intriguingly, suppressing caspase-8 is associated with increased sensitivity of cancer cells to DNA-damaging compounds and, as already pointed out, CDDP is a DNA intercalating agent (Fianco et al. 2018).

In response to chemotherapeutics’ action, the intrinsic pathway of apoptosis can be activated (Wang and Youle 2009). This pathway is mediated by Bax protein whose activation induces mitochondrial membrane permeabilization and cytochrome c release (Peña-Blanco and García-Sáez 2018). Furthermore, cytochrome c associates with Apaf-1 and procaspase-9 and generates the apoptosome that induces the activation of caspase-9. Then, caspase-9 activates caspase-3 and induces apoptosis (Jan and Chaudhry 2019).

In our study, activation of caspase-9 was detected only in the cells in 2D cultures, after 48 h of treatment with the complex, which highlights the role of the mitochondrial pathway in the apoptosis of breast cancer cells. Moreover, the involvement of this pathway in apoptosis of MDA-MB-231 cells exposed to SWCNT–COOH–CDDP treatment could be sustained also by the decrease of mitochondrial membrane potential (MMP) (Ly et al. 2003). The decline of MMP leads to condensation of the mitochondrial matrix and can be achieved by several mechanisms based on the deficiency of oxidizable substrates for the mitochondria, blockage of respiration, or uncoupling of the inner membrane (Gottlieb et al. 2003). In 2D cultures, cell death was induced by both intrinsic and extrinsic pathways, as suggested by caspase-8 and caspase-9 protein expressions. The activation of these pathways could be interconnected by Bid protein. Caspase-8 can activate Bid protein which is further translocated to the mitochondria where induces the release of cytochrome c and activation of the intrinsic pathway (Jan and Chaudhry 2019).

**Fig. 13:**
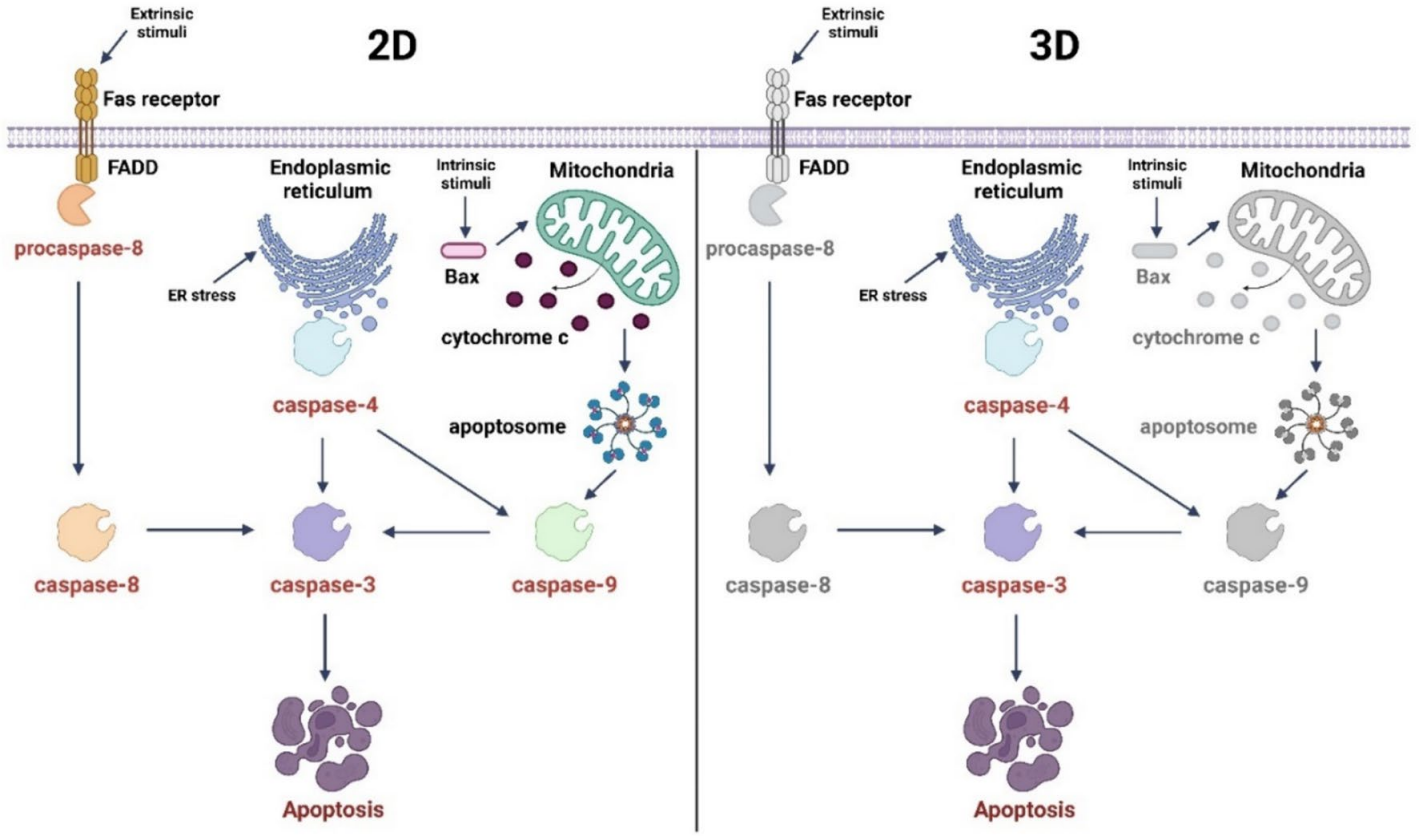
Schematic representation of apoptosis signaling pathways triggered in 2D and 3D-spheroid triple-negative breast cancer cultures by carboxyl-functionalized single-walled carbon nanotubes complexed with cisplatin. In red are marked the activated proteins of this targeted signaling pathway, while in black are highlighted proteins whose expressions were not assessed in the study. In gray are marked proteins that were not activated. Created with BioRender.com

Because in 3D cultures the expression of caspase-3 was up-regulated after the treatment with the complex, lacking the activation of caspase-8 or caspase-9, it was explored an alternative pathway of apoptosis mediated by endoplasmic reticulum (ER) stress. Caspase-4 is localized to the outer membrane of ER and mediates the ER stress-induced apoptosis, a mechanism which is poorly described (Gao and Wells 2013). It acts as an initiator caspase that activates caspase-3 or caspase-9 and trigger apoptosis (Heath-Engel et al. 2008; Guo et al. 2016). After treatment with SWCNT–COOH–CDDP complex, caspase-4 was activated in both 2D and 3D breast cancer cultures, which can highlight the initiation of ER stress-induced apoptosis. Considering the activation of caspase-4, caspase-3, and caspase-9 in MDA-MB-231 cells, it could be postulated two pathways of caspase-3 cleavage. First, caspase-4 activated caspase-9 which further induced the activation of caspase-3 which triggered apoptosis or, on the other way, caspase-4 and caspase-9 operated in two distinct signaling pathways and both culminated with caspase-3 cleavage and apoptosis. Instead, in MDA-MB-231 spheroids, the results could suggest a direct cleavage of caspase-3 by caspase-4, in response to ER stress (Fig. 13). The mechanisms involved in the caspase-4 activation are less defined, but it is known that this protein requires dimerization and interdomain processing and can be activated by calpains (Heath-Engel et al. 2008).

Invasion into the surrounding tissues is the first step of cancer cells’ metastasis process and is based on the detachment of invasive cells from the tumor mass. Further, the invasive cells are able to penetrate the basement membrane and invade the surrounding tissues (Gerashchenko et al. 2019). As the human adenocarcinoma MDA-MB-231 cell line is highly invasive (Mohammed et al. 2020), we assessed how the invasion potential of cells cultured in 2D and 3D systems was influenced after treatment exposure. The inhibition of invasion potential by SWCNT–COOH–CDDP in both 2D and 3D cultures (both doses and intervals) and by CDDP in 2D cultures (24 h, both doses and 48 h, low dose) could demonstrate the capacity of the samples to suppress the metastasis of breast cancer cells. This time, the effects induced by the SWCNT–COOH–CDDP sample in 2D cultures were not strongly different compared to 3D cultures. It is possible that the results were influenced by the utilization of different systems for the assessment of invasion, the one for 2D cultures was based on a polycarbonate membrane, and the one for 3D cultures was based on a hydrogel network. However, the increase of invasion potential induced by CDDP (both doses) and SWCNT–COOH (low dose) in 3D cultures could indicate the stimulation of cell capacity to metastasize. The variation of invasion potential after treatment could be correlated with the proliferative phenotype of both 2D and 3D cultures indicated by MCM2 protein expression (Gao et al. 2005). The inhibition of invasion capacity of the MDA-MB-231 cells could occur by various mechanisms, such as suppression of nuclear factor-κB-dependent matrix metalloproteinase-9 expression (Zhai et al. 2015) or suppression of matrix metalloproteinase-2 and -9 by PI3K/Akt signaling (Zhou et al. 2014). Another mechanism can be represented by epithelial–mesenchymal transition which is based on the loose of cell polarity and intercellular adhesion of epithelial cells, gained migration, and invasiveness and interstitial cell transition (Yang et al. 2021).

Overall, MDA-MB-231 cells cultured in 2D monolayers showed different drug responses compared to the ones cultured in a 3D system, which were less sensitive to CDDP action. This effect could be associated with the presence of the extracellular matrix in the case of spheroids. Matrigel is a basement membrane composed of laminin, collagen IV, heparan sulfate proteoglycans, entactin/nidogen, and growth factors which can offer a protected environment of tumor cells to drug action (Fridman et al. 1990). The influence of extracellular matrix (ECM) in the modulation of cancer cells' response to drugs was mentioned in other studies (Jurj et al. 2022; Cortini et al. 2023). Moreover, the higher resistance of 3D cultures compared to monolayer ones could be also explained by hypoxia development in spheroids as a result of limited diffusion of oxygen to the spheroid center. Tumor cells adapt to a hypoxic environment by activation of DNA damage repair proteins and metabolism alteration (Hamilton and Rath 2019), and hypoxia is frequently associated with resistance to chemotherapy treatment (Däster et al. 2017). Furthermore, as hypoxic niches can alter drug uptake, the responsiveness of 3D cultures can also be linked to limited drug penetration (Cosse and Michiels 2008).

Taken together, the significant results revealed a high anti-tumor activity of the complex in both 2D and 3D cultures, compared to free CDDP, and the potential to activate different apoptosis pathways in MDA-MB-231 cells and spheroids, highlighting the great relevance of the experimental culture model in determining the anti-cancer action of various drug complexes in preclinical studies. Also, our findings emphasize the great efficiency of carbon nanotubes in improving the drug response in cancer therapy and support the hypothesis that 3D cultures might represent a better drug screening platform that could provide more clinically relevant results.

Besides the important findings revealed by our study, the experimental setup could be associated with minor limitations linked to the experimental model. More advanced 3D models based on co-culture spheroids of cancerous cells—fibroblasts or normal cells, or perfused 3D systems are already used in the investigation of cancer therapies, as more relevant experimental cultures that offer a better approximation of the drug response induced in vivo.

## Conclusions

In this work, the effects of SWCNT–COOH–CDDP and free components toward 2D and 3D-spheroid cultures of triple-negative breast cancer cells were compared. We have demonstrated that chemotherapeutic drugs can activate different cell death mechanisms depending on the in vitro experimental culture model used. The results revealed a high anti-tumor activity of the complex, in both 2D and 3D models, suggesting its improved therapeutic potential. Our data showed that this enhanced therapeutic effect was mediated by the reduction of cell viability and proliferation, membrane permeabilization, and occurrence of oxidative stress. More detailed biological assessments demonstrated activation of apoptosis in both 2D and 3D cultures of breast adenocarcinoma by a dose of 0.632 μg/mL SWCNT–COOH–CDDP. However, the complex activated different pathways of apoptosis: synergistic action of extrinsic, intrinsic, and endoplasmic reticulum pathways in 2D cultures and endoplasmic reticulum stress-induced apoptosis in spheroids. Overall, the spheroids presented higher resistance to treatment action, compared to 2D cultures, as indicated by membrane integrity assessment, proliferation markers, or executioner caspase-3 expression. Generally, this study offered an overview of the cell death mechanisms activated by CDDP in breast cancer cells and showed that SWCNT–COOH–CDDP seems to be a promising treatment approach for triple-negative breast cancer treatment. Moreover, the relevance of using 3D-spheroids in the in vitro screening of drug formulations was proved.

In future, the investigation of SWCNT–COOH–CDDP effects on normal breast cancer cells will be pursued to further disclose and optimize the potential of the complex for triple-negative breast cancer treatment.

## Abbreviations

MCTSs: Multicellular tumor spheroids
CDDP: Cisplatin
SWCNT–COOH: Single-walled carbon nanotubes functionalized with carboxyl groups
SWCNT–COOH–CDDP: Single-walled carbon nanotubes functionalized with carboxyl groups and conjugated with cisplatin
LDH: Lactate dehydrogenase
GSH: Reduced glutathione
MMP: Mitochondrial membrane potential
ER: Endoplasmic reticulum
PVDF: Polyvinylidene fluoride
